# Biomaterials in Postoperative Adhesion Barriers and Uterine Tissue Engineering

**DOI:** 10.3390/gels11060441

**Published:** 2025-06-09

**Authors:** Abbas Fazel Anvari-Yazdi, Ildiko Badea, Xiongbiao Chen

**Affiliations:** 1 Division of Biomedical Engineering, University of Saskatchewan, 57 Campus Dr, Saskatoon, SK S7K 5A9, Canada; 2 College of Pharmacy and Nutrition, University of Saskatchewan, Saskatoon, SK S7N 5E5, Canada; ildiko.badea@usask.ca; 3 Department of Mechanical Engineering, University of Saskatchewan, 57 Campus Dr, Saskatoon, SK S7K 5A9, Canada

**Keywords:** bioprinting, electrospinning, biomaterials synthesis, biopolymers

## Abstract

Postoperative adhesions (POAs) are a common and often serious complication following abdominal and gynecologic surgeries, leading to infertility, chronic pain, and bowel obstruction. To address these outcomes, the development of anti-adhesion barriers using biocompatible materials has emerged as a key area of biomedical research. This article presents a comprehensive overview of clinically relevant natural and synthetic biomaterials explored for POA prevention, emphasizing their degradation behavior, barrier integrity, and translational progress. Natural biopolymers—such as collagen, gelatin, fibrin, silk fibroin, and decellularized extracellular matrices—are discussed alongside polysaccharides, including alginate, chitosan, and carboxymethyl cellulose, focusing on their structural features and biological functionality. Synthetic polymers, including polycaprolactone (PCL), polyethylene glycol (PEG), and poly(lactic-co-glycolic acid) (PLGA), are also examined for their tunable degradation profiles (spanning days to months), mechanical robustness, and capacity for drug incorporation. Recent innovations, such as bioprinted and electrospun dual-layer membranes, are highlighted for their enhanced anti-fibrotic performance in preclinical studies. By consolidating current material strategies and fabrication techniques, this work aims to support informed material selection while also identifying key knowledge gaps—particularly the limited comparative data on degradation kinetics, inconsistent definitions of ideal mechanical properties, and the need for more research into cell-responsive barrier systems.

## 1. Introduction

Postoperative adhesions (POAs)—fibrous bands of scar tissue forming between organs after surgery—are a prevalent and serious complication across nearly all surgical fields and are inevitable. Despite advances in surgical technique, adhesions still develop in 50–95% of patients after abdominal or pelvic operations [1,2]. In gynecological surgery, adhesion formation is of particular concern, as it often leads to chronic pelvic pain, bowel obstruction, and infertility [3]. Concurrently, injuries to the uterus (from obstetric procedures, infection, or curettage) can result in intrauterine adhesions (Asherman’s syndrome) or uterine wall defects, which compromise reproductive function and pregnancy outcomes [4].

One promising solution to postoperative adhesion is using barrier materials that temporarily separate injured tissue surfaces during the critical early phase of wound healing, typically the first 3 to 7 days after surgery, when fibrin deposition and fibroblast proliferation drive adhesion formation [2]. These barriers can be fabricated from biocompatible materials that degrade safely after fulfilling their function. Simultaneously, advances in tissue engineering enable the development of biomaterial scaffolds that mimic the native extracellular matrix of the uterus, providing a supportive environment for cell attachment, proliferation, and differentiation.

A wide range of materials have been investigated for these applications. Natural biopolymers such as collagen, gelatin, fibrin, silk fibroin, and decellularized extracellular matrix (dECM) offer high biocompatibility and bioactivity [5,6]. Polysaccharides like cellulose, chitosan, and alginate contribute useful properties such as gelation, antimicrobial activity, and ease of modification [7,8]. Synthetic polymers such as polycaprolactone (PCL), polyethylene glycol (PEG), and poly(lactic-co-glycolic acid) (PLGA) allow for the fine-tuning of mechanical strength and degradation kinetics [9,10]. Non-degradable polymers like ePTFE and polypropylene, while less commonly used today due to their permanence, have played a key role in the history of surgical biomaterials and are still applied in cases requiring permanent structural reinforcement [11].

In addition to material composition, fabrication techniques play a pivotal role in determining the performance of biomaterial systems. Conventional methods such as solvent casting, gas foaming, porogen leaching, phase separation, and melt molding are employed to control porosity, mechanical integrity, and degradation behavior. For example, gas foaming and porogen leaching are widely used to create interconnected porous structures essential for tissue scaffolds, while phase separation and melt molding are leveraged for their ability to produce custom-shaped and load-bearing constructs.

This paper aims to review the current state of biomaterials used in these two applications—postoperative adhesion barriers and uterine scaffolds—focusing on the design criteria, fabrication strategies, and functional outcomes. By reviewing existing research and identifying gaps in knowledge, we hope to offer insights into the development of next-generation biomaterials that are not only safer and more effective, but also tailored to the complex requirements of female reproductive health and surgical recovery, including smart materials with bioresponsive degradation, targeted drug release, and tissue-regenerative capabilities.

## 2. Biomaterials to Fabricate Anti-Adhesion Barriers

In recent decades, biocompatible and biodegradable polymers have emerged as crucial tools in preventing POAs by serving as physical barriers to inhibit tissue adhesion following surgery. These anti-adhesion biomaterials must meet several essential criteria to achieve optimal effectiveness, including superior biocompatibility and non-toxicity, which ensure that the material does not trigger adverse immune responses or inflammation. Controlled biodegradability is also vital, as the biomaterial should degrade at a rate that aligns with the critical healing period without leaving harmful byproducts. It is equally crucial to consider the mechanical and structural aspects of these biomaterials. They must possess adequate strength and flexibility to conform to tissue surfaces and remain intact during the healing process. Moreover, their ease of application plays a critical role in surgical settings, where materials that are simple to use—such as films, sprays, or hydrogels—can significantly reduce operative time and complexity [12,13,14].

One of the primary goals of these materials is to avoid fibrin bridges forming, as these bridges serve as precursors to adhesions by providing a scaffold for fibroblast proliferation and collagen deposition [15]. Biomaterials are designed to promote controlled healing by supporting tissue regeneration while minimizing excessive fibrotic activity to further enhance efficacy. They must also work to prevent inflammatory responses, as prolonged inflammation can exacerbate adhesion formation [16].

Both natural and synthetic polymers have been extensively studied and employed for these purposes, each offering distinct advantages. Natural polymers, derived from biological sources, are inherently biocompatible and promote excellent tissue integration. However, they often exhibit weaker mechanical properties, batch-to-batch variability, and scalability challenges [17,18]. In contrast, synthetic polymers offer greater versatility, enabling tunable biodegradation rates and superior mechanical stability [19]. They can also be functionalized for therapeutic agent delivery, addressing multiple needs simultaneously.

In the following sections, we will explore the various biomaterials utilized in anti-adhesion barrier applications, with a focus on their specific roles in POA prevention.

### 2.1. Natural Biopolymers in Adhesion

#### 2.1.1. Proteins

##### Collagen

Collagen is the most abundant protein, making up about 20–30% of the total protein in the human body, and plays a crucial role in the structural integrity of various tissues. It is a fibrous protein that provides strength and elasticity to connective tissues and the extracellular matrix (ECM) [6]. Collagen possesses unique properties that make it highly suitable for anti-adhesion applications, including excellent biocompatibility, biodegradability, hydrophilicity, and non-immunogenicity, in addition to its ability to act as a physical barrier against fibrin bridge formation [20,21]. There are various types of collagens, with types I, II, III, and IV being the most prevalent in the body. Among these, type I collagen has been extensively utilized in anti-adhesion barriers due to its structural stability and capacity to modulate tissue repair processes [22].

**Figure 1:**
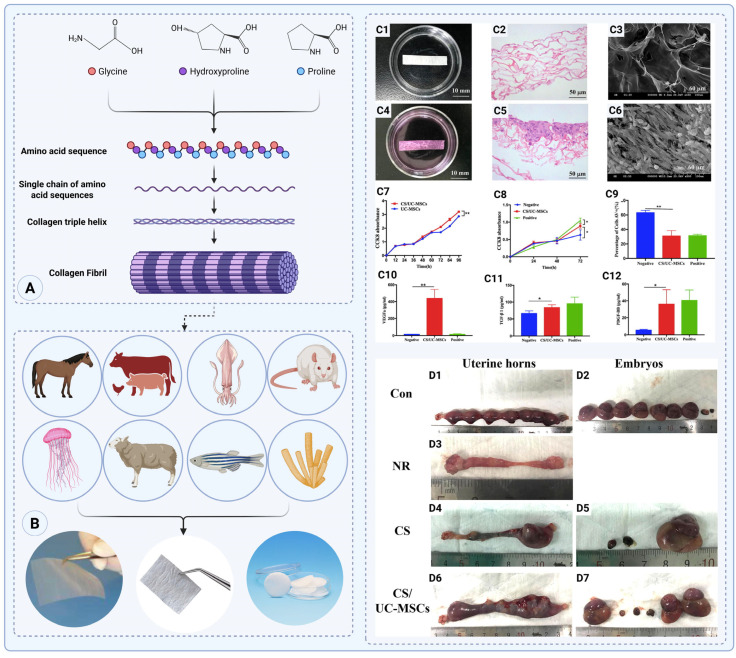
**Collagen in anti-adhesion barriers and endometrial regeneration.** (**A**) Structural representation of collagen. (**B**) Sources of collagen from animal and marine origins and applications of animal- and marine-derived collagens in anti-adhesion barrier system in uterine reconstruction (created with Biorender.com). (**C1**–**C6**) Morphological and histological characterization of the collagen scaffold (CS) before and after UC-MSC seeding, showing porous structure, effective cell attachment, and proliferation. (**C7**–**C9**) UC-MSCs enhanced HESC proliferation and reduced apoptosis in co-culture. (**C10**–**C12**) ELISA confirmed elevated secretion of pro-regenerative factors (VEGF-A, TGF-β1, PDGF-BB). (**D1**–**D7**) Gross views of uteri and embryos indicate restored fertility in the CS/UC-MSC group, with higher implantation rates compared to control groups (reproduced with permission, Elsevier). The significance levels are indicated as follows: ** for *p* < 0.01 and * for *p* < 0.05.

Collagen molecules are composed of three polypeptide chains that form a triple-helix structure, which assembles into fibrils and further aggregates into larger fibers (Figure 1A). Collagen can be extracted from various animal sources, such as bovine [23], porcine [24], equine [25,26], poultry [27], rat tails [28], and sheep skin [29], as well as marine sources like jellyfish, squid, and sponges [30,31] (Figure 1B). Marine collagen peptides, produced through enzymatic or chemical hydrolysis, possess low molecular weight, enhanced solubility, and high bioavailability. These features make them promising for biomedical applications. A recent in vivo study showed that fish-collagen-based surgical compresses significantly accelerated wound closure while reducing local inflammation, suggesting that marine collagen creates a pro-healing environment conducive to orderly tissue regeneration [32]. These characteristics—namely, inflammation modulation and supportive healing—are essential for developing anti-adhesion biomaterials aimed at minimizing fibrotic bridging post-surgery [23,30,33]. This increased solubility enhances their functionality in medical applications, particularly in anti-adhesion films, as they can be more easily processed and integrated into formulations like gels or films (Figure 1B).

In a multicenter, randomized study, 150 women underwent hysteroscopic surgery and received a type I collagen gel (Collabarrier^®^,Dalim Tissen Co., Ltd., Hwaseong-si, Gyeonggi-do, Republic of Korea) applied inside the uterus, compared to the standard hyaluronate–carboxymethylcellulose (HA/CMC) gel. Second-look hysteroscopy at 1 month showed no significant differences in intrauterine adhesion incidence or severity between the collagen and HA/CMC groups [34]. The trial was limited to Korean patients and relied on a photographic assessment of adhesions, which may underestimate small synechiae. The collagen gel’s efficacy was comparable to the standard treatment, suggesting that it can be effectively used to minimize adhesions in reproductive surgery.

In a rat model of severe intrauterine adhesion (IUA), the application of a collagen scaffold (CS) loaded with human umbilical-cord-derived mesenchymal stem cells (UC-MSCs) significantly promoted endometrial regeneration and reduced adhesion recurrence [35]. The collagen scaffold (CS), fabricated via freeze drying and thermal crosslinking, exhibited a porous architecture with pore sizes of 100–200 µm, providing a favorable 3D environment for UC-MSC adhesion and proliferation. When seeded with UC-MSCs, the CS supported significantly enhanced cell viability compared to tissue culture polystyrene (TCPS), with CCK-8 absorbance values after 72 h reaching ~0.90 vs. ~0.75 on TCPS (*p* < 0.05). In co-culture with human endometrial stromal cells (HESCs), the CS/UC-MSC construct promoted HESC proliferation (OD450 = 0.90 ± 0.04 vs. 0.60 ± 0.09 in the negative control, *p* < 0.05) and reduced apoptosis to 31 ± 4%, significantly lower than the control (62 ± 2%, *p* < 0.01). The ELISA analysis of co-culture supernatants revealed substantial paracrine factor secretion: VEGF-A increased to 440 ± 50 pg/mL vs. 15.2 ± 0.9 pg/mL (*p* < 0.01), TGF-β1 to 84 ± 4 pg/mL vs. 66 ± 5 pg/mL (*p* < 0.05), and PDGF-BB to 36 ± 8 pg/mL vs. 5.4 ± 0.4 pg/mL (*p* < 0.05) (Figure 1(C1–C12)). In vivo, the transplantation of CS/UC-MSCs into a rat model of intrauterine adhesion (IUA) led to significantly improved endometrial regeneration. At 60 days post-transplantation, the endometrial thickness in the CS/UC-MSC group reached 610 ± 30 µm, markedly greater than that in the CS (220 ± 20 µm) and NR (170 ± 20 µm) groups (*p* < 0.01). Collagen deposition was also significantly reduced to 41 ± 2%, comparable to the sham group (41 ± 3%) and far lower than in the CS (74 ± 2%) and NR (75 ± 2%) groups (*p* < 0.01). Immunohistochemical staining confirmed increased cell proliferation (Ki67+: 21 ± 2%) and epithelial regeneration (pan-cytokeratin+: 26 ± 1%) in the CS/UC-MSC group by day 60, reaching levels statistically similar to the sham group (*p* > 0.05). Furthermore, the expression of ERα and PR was significantly restored, reaching 15.3 ± 0.9% and 19 ± 2%, respectively, vs. ~4–5% in the CS and NR groups (*p* < 0.01). Functionally, the CS/UC-MSC group achieved a 45.0% pregnancy rate and a 30.3% embryo implantation rate, while no pregnancies occurred in the NR group and only 5.0% were observed in the CS group. These findings demonstrate that the CS/UC-MSCs system not only supports the structural regeneration of the endometrium, but also restores its functional competence, offering a potent strategy for treating IUA and promoting fertility restoration (Figure 1(C1–C12, D1–D7)) [35].

Beyond this, synthetic collagen is also being explored as a promising option for tissue engineering applications [36]. Collagen mimetic peptides (CMPs) and recombinant human collagen (rhCollagen) offer the benefit of reduced immunogenicity and antigenicity compared to animal-derived collagen, making them less likely to provoke immune responses [37,38,39]. Notably, plant-derived rhCollagen has shown no antigenic or immunogenic responses in human CD4^+^ T cells, in contrast to bovine collagen, which is known to trigger immune reactions [40]. This makes rhCollagen particularly suitable for patients sensitive to traditional collagen sources or those at higher risk of foreign body reactions.

RhCollagen, in particular, represents a significant advancement in the field of biomaterials [41]. Its ability to be produced without animal-derived contaminants addresses safety concerns and offers increased versatility in medical applications. Researchers are increasingly exploring the potential of hybrid films that combine synthetic collagen with other biodegradable materials, such as hyaluronic acid (HA) or carboxymethyl cellulose (CMC), to improve the mechanical strength and durability of anti-adhesion barriers [9,42,43,44].

##### Gelatin

**Figure 2:**
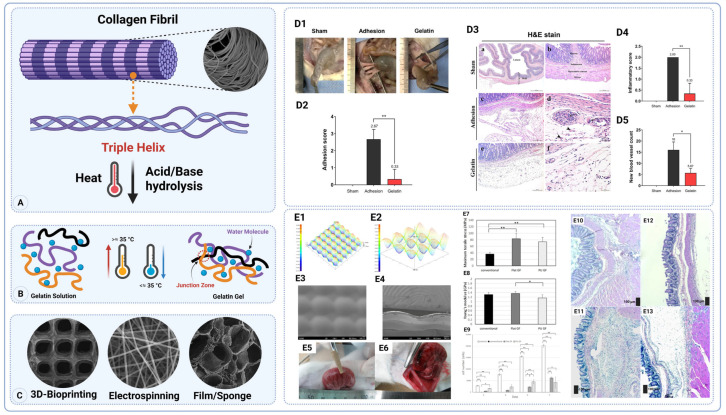
(**A**) Collagen triple-helix acid and base hydrolysis to break down bonding to form gelatin. (**B**) Triple helices supported by intermolecular hydrogen bonds cause a gelatin solution to gel when cooled; heating the resultant gelatin gel causes the opposite effect. (**C**) Application of gelatin as a biomaterial (created with Biorender.com). (**D1**–**D5**) Macroscopic and histological assessment of abdominal adhesions 14 days post-surgery. (**D1**) Representative images of sham, adhesion, and gelatin groups. (**D2**) Adhesion scores (0–4 scale) show reduced adhesions in the gelatin group. (**D3 a-f**) Adhesion tissues from the sham, adhesion, and gelatin groups were stained with H&E. Newly formed blood vessels (arrowheads) and multinucleated giant cells (arrows) are visible. Scale bars: (**A**) a = 500 μm; b, c, e = 100 μm; d, f = 20 μm. (**D4**) Inflammatory scores (0–3 scale) and (**D5**) neovessel counts confirm lower inflammation and vascularization in the gelatin group (reproduced with permission, Elsevier) [48]. (**E1**–**E13**) Characterization and evaluation of PU GF anti-adhesion film. (**E1**–**E4**) Surface topography and structure of PU GF by roughness profiling and SEM imaging. (**E5**–**E6**) Rat cecum abrasion model showing injury sites and sutured adhesion setup. (**E7**–**E9**) PU GF exhibits superior tensile strength, elasticity, and enhanced fibroblast proliferation over time. (**E10**–**E13**) H&E-stained sections reveal reduced inflammation and fibrotic response with Flat GF and PU GF compared to control and conventional films (Copyright: © 2025 Ozamoto et al. (PLoS One), licensed under CC BY 4.0) [49]. The significance levels are indicated as follows: ** for *p* < 0.01, * for *p* < 0.05.

Gelatin, a natural polymer derived from collagen, has a long history of use in medical and biological applications owing to its biocompatibility and degradability. Gelatin is produced by the partial acidic, alkaline, pepsin, and trypsin hydrolysis of collagen, a fibrous protein found in animals’ extracellular matrix (ECM) [45]. During this process, collagen’s highly ordered structure is broken down, yielding gelatin, which maintains some of the bioactive properties of collagen but in a denatured, water-soluble form (Figure 2A–C) [46]. The resulting gelatin is a polypeptide that retains many of the original collagen bioactive motifs [47].

Gelatin can be processed into various forms, including hydrogels, sponges, and films [50,51]. However, due to the denaturation process, gelatin has weaker mechanical strength and is much more thermosensitive, meaning it loses its structural integrity at body temperature unless chemically modified or crosslinked [51,52].

This limitation becomes especially critical in dynamic or load-bearing environments—such as sites with frequent organ movement (e.g., the intestines or uterus)—where mechanical stability is essential for effective anti-adhesion performance. A recent in vivo study demonstrated the strong anti-adhesive potential of a commercially available gelatin sponge (GELITA-SPON^®^ STANDARD, GELITA MEDICAL GmbH, Eberbach, Germania) in a rat model of post-surgical intra-abdominal adhesions [48]. Application of the gelatin sponge directly onto the abraded cecum reduced adhesion formation by 91% compared to untreated controls. Histological and histochemical analyses revealed significantly decreased inflammatory cell infiltration, neovascularization, and collagen fiber deposition in the gelatin group (*p* < 0.001). Importantly, immunohistochemical staining showed a marked increase in matrix metalloproteinase-9 (MMP-9) expression and a decrease in macrophage marker (MAC387), suggesting enhanced fibrinolytic activity and the suppression of inflammation as key mechanisms. No adverse effects were observed, and the sponge was fully resorbed by day 14 post-surgery. These findings support the use of absorbable gelatin sponges as safe, bioactive physical barriers capable of modulating the postoperative healing environment to prevent intra-abdominal adhesions (Figure 2(D1–D5)) [48]. This animal study demonstrates dramatic efficacy, but only short-term outcomes in rodents were assessed. Further studies are needed to ensure that sponges fully resorb and to evaluate functional recovery before clinical use.

A newly developed punctate-structured gelatin film (PU GF), derived from porcine type I collagen and thermally crosslinked, demonstrated superior anti-adhesion performance and handling characteristics compared to both a conventional hyaluronic acid/carboxymethyl cellulose (HA/CMC) film and a flat gelatin film (Flat GF) [49]. In a rat cecum abrasion model, PU GF significantly reduced adhesion extent and severity (0.71 ± 1.50 and 0.43 ± 0.79, respectively) compared to the controls (3.88 ± 0.35 and 3.63 ± 0.74, *p* < 0.05), and maintained efficacy even after re-attachment. PU GF also exhibited enhanced mechanical properties, including high tensile strength (73.83 ± 13.35 MPa) and ductility (fracture strain 7.27 ± 2.5%), and superior adhesion to tissue (4.0 ± 0.76 MPa) while minimizing undesirable self-adhesion and silicon adherence. Unlike the conventional film, which completely inhibited fibroblast proliferation, PU GF supported moderate, non-cytotoxic cell growth, suggesting a safer mechanism of adhesion prevention via controlled degradation rather than cytotoxicity. The gelatin film’s textured surface improved flexibility and reduced self-sticking, allowing repositioning (“re-attachment”) during surgery. Together, these findings highlight PU GF’s potential as a practical, bioactive, and re-attachable anti-adhesion barrier suited for laparoscopic applications (Figure 2(E1–E13)). Table 1 highlights the advantages and disadvantages of using gelatin in POA applications.

##### Fibrin

**Figure 3:**
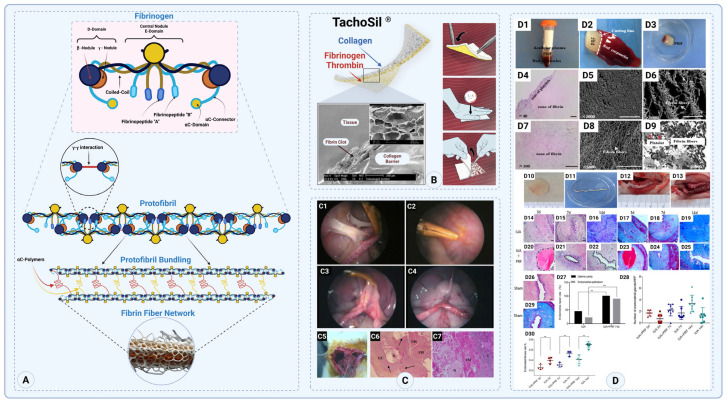
(**A**) Fibrinogen assembly structure (created with Biorender.com). (**B**) TachoSil^®^ (Takeda Pharmaceutical Company, Ltd., Osaka, Japan) structure. (**C**) Postoperative and histological evaluation of intrauterine adhesions. (**C1**–**C4**) Laparoscopic views showing varying adhesion severity, from dense uterine adhesions to adhesion-free healing. (**C5**) Laparotomic view during hysterectomy and peritoneal biopsy in 49 rats/group to assess early effects of TachoSil^®^. (**C6**–**C7**) Histology shows reduced uterine fibrosis and inflammation with TachoSil^®^, but no peritoneal improvement due to bipolar coagulation injury (UH uterine horn, UC uterine corpus, N necrosis, arrows inflammation) (adapted with permission, Springer) [67]. (**D1**–**D30**) Evaluation of PRF structure and its effects on endometrial repair in a rat IUA model. (**D1**–**D9**) PRF showed a distinct fibrin network and platelet-rich zones by histology, SEM, and TEM. (**D10**–**D30**) PRF transplantation promoted epithelial regeneration, increased gland numbers, and reduced fibrosis compared to the IUA group, with outcomes approaching the sham group by day 14 (Copyright: © 2023 Mao et al. (Frontiers), licensed under CC BY 4.0) [68]. The significance levels are indicated as follows: *** for *p* < 0.001, ** for *p* < 0.01 and * for *p* < 0.05.

Fibrinogen is a glycoprotein produced in the liver and released into circulation, which is essential for various biological functions, including hemostasis, wound healing, inflammation, and angiogenesis [64]. Fibrinogen is a fibrous, dimeric protein roughly 45 nm long, with a molecular weight of 340 kDa and consisting of around 132 amino acids. The molecule exhibits symmetry, with two identical areas termed D-domains, interconnected by a central E-domain. The hexameric structure has three unique pairs of polypeptide chains—α, β, and γ. The naming of these chains, as elucidated by Weisel et al., originates from the fibrinopeptides A and B, which are cleaved from fibrinogen by thrombin to produce fibrin, while the residual polypeptide chains are designated as α and β [65]. The γ chains remain unaltered, since thrombin does not break them. The D-domains are linked to the E-domain by five symmetrical disulfide bridges, forming a coiled-coil structure (Figure 3A) [66].

Fibrin has gained much attention because of its potential in adhesion barrier applications due to its biocompatibility, biodegradability, and versatile properties [69]. Fibrin is a significant naturally occurring protein throughout blood clotting. Fibrin also plays a central role in wound healing and tissue repair, making it an attractive biomaterial as an adhesive for wound closure in various applications [70].

Fibrin glue is effective in controlling bleeding during surgery, providing a valuable tool for surgeons in managing intraoperative hemostasis. Fibrin’s role in wound healing extends beyond its use as a sealant. Fibrin-based dressings are utilized in chronic wound management due to their biologically active matrix that promotes healing. However, their use as standalone adhesion barriers is limited because fibrin can contribute to adhesion formation [71,72,73]. To address this, combinations of fibrin with other biomaterials have been explored. One of the earliest clinically evaluated hemostatic and anti-adhesion barriers was TachoSil^®^, a sponge-like sheet composed of equine collagen coated with human fibrinogen and bovine thrombin (Figure 3B). When moistened with saline, the coated side formed a fibrin barrier at the application site, enabling effective hemostasis while acting as a physical barrier to prevent postoperative adhesions. In a study involving 16 gynecological surgery patients, complete hemostasis was achieved in 87.5% of cases, and no de novo adhesions were observed at application sites upon second-look laparoscopy or laparotomy conducted up to 4 years post-operation. TachoSil^®^ was fully resorbed in most patients, and no adverse tissue reactions were reported, highlighting its dual efficacy as both a hemostatic agent and a long-term anti-adhesion barrier [74]. In another clinical setting, TachoSil^®^ significantly reduced postoperative adhesion formation, with one referenced trial showing that only 2 out of 22 patients (9%) treated with TachoSil had postoperative adhesions, compared to 13 out of 23 patients (57%) in the control group (*p* < 0.01) [75].

In a randomized, controlled laparoscopic rat model, TachoSil^®^, a fibrinogen- and thrombin-coated collagen sponge, significantly reduced postoperative adhesion formation [67]. Following standardized injury to the uterine horn, corpus, and peritoneum, the TachoSil^®^ application resulted in markedly lower adhesion scores compared to untreated controls: 12.96 vs. 21.66 for the peritoneum, 7.22 vs. 15.20 for the uterine horn, and 5.88 vs. 34.52 for the uterine corpus. Histological evaluation further revealed that TachoSil^®^ minimized tissue fibrosis and inflammation, indicating both a mechanical barrier effect and biological modulation of healing responses. These results support its dual role in hemostasis and effective adhesion prevention (Figure 3(C1–C7)) [67].

Adhexil™ (Omrix Biopharmaceuticals, Ltd., Tel Aviv, Israel), a fibrin sealant enhanced with tranexamic acid, is a notable achievement in uterine wound healing products, exhibiting superior efficacy in significantly reducing postoperative adhesions, as shown by compelling evidence from both preclinical and clinical studies [76]. In a rabbit uterine horn model, Adhexil™ significantly reduced adhesion formation compared to other barriers such as Seprafilm™ (Genzyme Corporation, Deerfield, IL, USA) and Interceed™ (Ethicon, Inc., Raritan, NJ, USA). Additionally, a prospective, multicenter, randomized pilot clinical study evaluating Adhexil™ in women undergoing bilateral ovarian surgery reported a trend toward reduced incidence, severity, and extent of adhesions, supporting its potential utility in clinical settings [76]. Furthermore, a prospective, randomized, controlled study evaluated the adhesion-prevention effects of fibrin sealants after laparoscopic myomectomy [77]. The study found that the frequency of postoperative uterine adhesions was significantly lower in the fibrin gel group (34.5%) compared to the control group (62.5%) and the fibrin sheet group (67.7%). These findings suggest that fibrin gel may be effective in reducing postoperative adhesions following laparoscopic myomectomy [77].

A 2023 study conducted by Mao et al. using a rat model of intrauterine adhesion demonstrated that platelet-rich fibrin (PRF) transplantation resulted in a significant improvement in endometrial regeneration while concurrently decreasing the fibrotic area from approximately 25% to approximately 6%. In addition to other improvements, the rats treated with PRF also showed a significant increase in fertility, as evidenced by the increase in pregnancy rates from 16.7% in the untreated IUA group to 75% in the PRF-treated group. Further immunohistochemical analysis confirmed the enhanced proliferation and epithelial recovery observed, thus bolstering the promise of platelet-rich fibrin (PRF) as a valuable autologous therapeutic approach for the prevention of iatrogenic uterine perforation (IUA) and the subsequent restoration of endometrial tissue integrity, as evidenced by the findings presented in the study (Figure 3(D1–D30)) [68]. This benefit was shown in rodents; clinical studies are needed to confirm if intrauterine PRF can safely reduce adhesions in women. Additionally, PRF’s preparation must be standardized for reproductive use.

Consequently, a significant impediment to the broad adoption of fibrin-based barriers stems from the considerable challenges and expenses inherent in their large-scale manufacturing, considering the fibrin molecule’s origin in blood plasma [78,79]. In light of the considerable production obstacles encountered, investigations into the development of recombinant fibrinogen and the exploration of potential synthetic substitutes are currently underway [80].

##### Silk Fibroin

Silk fibroin (SF) is a natural protein biopolymer, primarily derived from the silk of the *Bombyx mori silkworm*, although other silk-producing organisms also generate fibroin proteins with similar structural properties [81]. SF has been of significant interest in various fields, especially biomedical research, due to its exceptional mechanical properties, biocompatibility, and biodegradability. Its hierarchical structure, versatility in processing, and adaptability for use in various forms—such as films, scaffolds, and hydrogels—have led to its increasing application in postoperative adhesion barriers [82].

**Figure 4:**
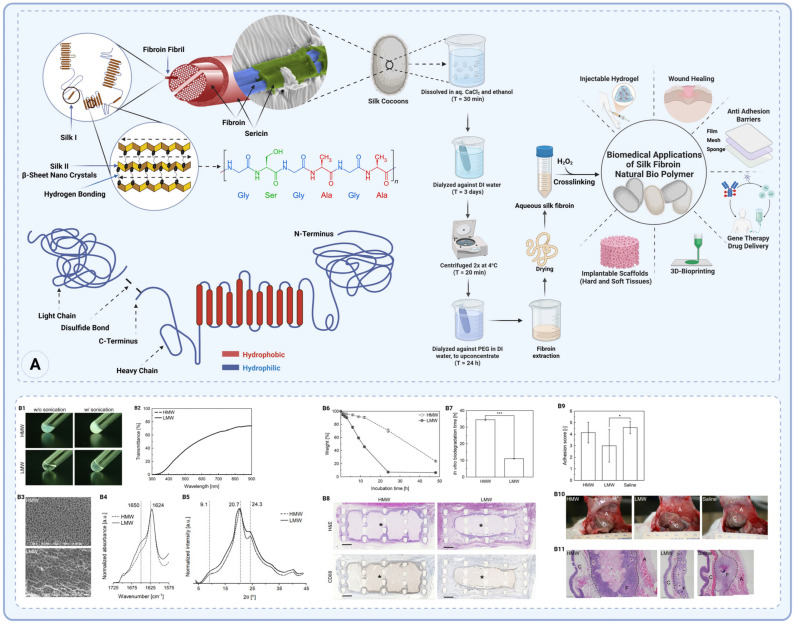
(**A**) Silk fibroin structure, synthesis, fabrication, and application as a natural biopolymer (created with Biorender.com). (**B1**–**B5**) Physical properties of HMW and LMW SF hydrogels, including gelation (**B1**), light transmittance (**B2**), SEM (**B3**), FTIR (**B4**), and XRD patterns (**B5**). B6–B8: Biodegradation analysis of SF hydrogels via in vitro (**B6**, **B7**) and in vivo (**B8**) studies, showing faster degradation in LMW SF (* *p* < 0.05, *** *p* < 0.0001). (**B9**–**B11**) Anti-adhesion effects of SF hydrogels in rats at day 7, with lower adhesion scores in LMW SF (**B9**), supported by gross (**B10**) and histological images (**B11**) (C, A, F, and asterisks indicate cecum, abdominal wall, fibrous tissue, and remaining SF hydrogels, respectively.) (reproduced with permission, ACS) [89].

SF consists of two primary protein chains: a heavy chain (H-chain) of 390 kDa and a light chain (L-chain) of 26 kDa, which are connected via disulfide bonds, forming an H-L complex. Additionally, the structure includes P25, a 25 kDa glycoprotein that contains Asn-linked oligosaccharide chains and is hydrophobically linked to the H-L complex (Figure 4A) [83]. In *Bombyx mori*, these three polypeptides—H-chain, L-chain, and P25—exist in a molar ratio of 6:6:1, respectively, to form the core silk protein [84,85]. The heavy chain is rich in glycine (45.9%), alanine (30.3%), serine (5.3%), valine (1.8%), and 15 other amino acids. The amino acid composition of SF, particularly in the heavy chain, features a high proportion of glycine (43%), alanine (30%), and serine (12%), which are integral to its structure and function [86,87,88].

The repetitive nature of the heavy chain, especially the Gly-X dipeptide motif (where X is often alanine or serine), accounts for 60–75% of the H-chain. Within this motif, hexapeptide sequences like Gly-Ala-Gly-Ala-Gly-Ser and dipeptide repeats such as Gly-Ala or Gly-Ala-Gly-Tyr contribute to the formation of stable anti-parallel β-sheet crystallites (Figure 4A) [90]. These β-sheets give silk fibroin its remarkable mechanical properties, such as tensile strength and toughness. In contrast, the L-chain, which has a non-repetitive amino acid sequence, is more hydrophilic and elastic compared to the H-chain, adding flexibility to the overall structure [91].

One of the key factors contributing to the widespread use of silk fibroin in anti-adhesion applications is its excellent biocompatibility. Silk fibroin has been approved by the FDA for medical applications, including sutures, since 1993 [92,93]. In vitro and in vivo studies have shown that SF supports cell adhesion, proliferation, and differentiation, making it an ideal substrate for scar healing. For instance, fibroblast cells have been successfully cultured on SF-coated films, demonstrating their compatibility with mammalian cells [94]. Additionally, SF has been shown to be compatible with blood, and scaffolds made from SF exhibit minimal immune response after implantation [95].

The rate of SF degradation can be influenced by its crystalline structure. Films with a higher proportion of silk I degrade more quickly than those with a higher silk II content, which is more resistant to degradation due to its stable β-sheet structure. In vivo studies have shown that SF scaffolds can degrade entirely within 18 to 36 months, depending on their morphology, with nanofibers degrading as quickly as 8 weeks [94,96]. SF is primarily degraded by proteolytic enzymes such as protease XIV, resulting in amino acids and small peptides that are resorbed or excreted via renal or hepatic pathways, depending on their molecular weight [97,98].

In a study, physical hydrogels of *Bombyx mori* silk fibroin were tested as absorbable adhesion barriers [89]. High-molecular-weight (HMW) silk hydrogels degrade slowly in vivo, and in a rat cecal abrasion model, a HMW silk film remained in the abdomen and did not reduce adhesions (adhesion severity equal to untreated controls). By contrast, an alkaline-treated low-molecular-weight (LMW) silk fibroin hydrogel, with reduced β-sheet crystallinity, fully biodegraded within ~2 weeks and significantly lowered adhesion severity scores vs. untreated rats. The LMW silk’s faster bioresorption was key: it elicited less fibrous encapsulation, lower plasma protein/fibroblast attachment, and was gone by the time healing occurred (Figure 4(B1–B11)) [89].

##### Complex Proteins/Decellularized Extracellular Matrix (dECM)

**Figure 5:**
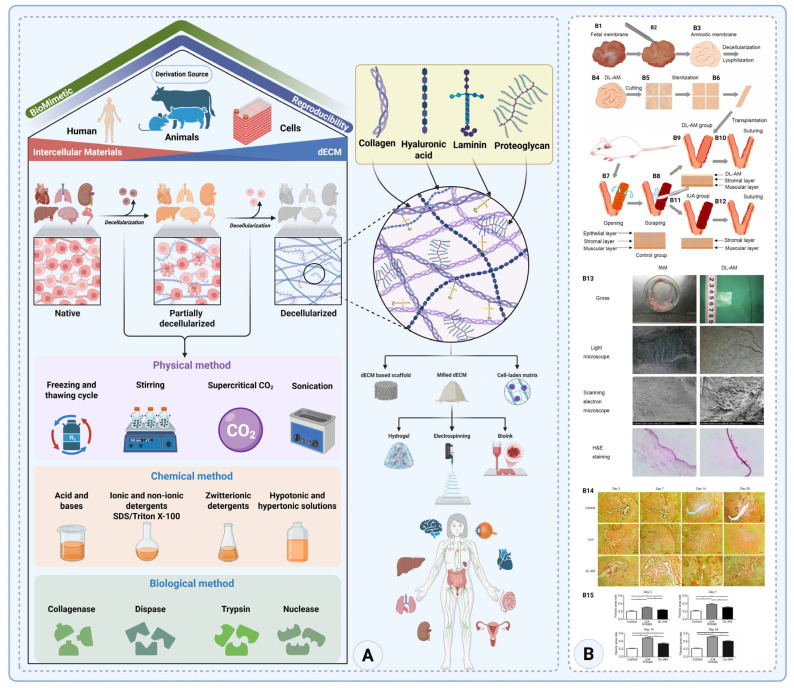
**Tissue engineering applications and decellularized ECM procurement.** (**A**) The secretive and ECM proteins found in the ECM promote cell division and proliferation. Alternative resources for tissue grafting are provided by the tissue structure and circulatory network that remain after the whole organ is decellularized. The dECM has many applications in different tissue engineering applications produced with different techniques, such as 3D-Bioprinting and electrospinning (created with Biorender.com). (**B1**–**B10**) Schematic of experimental procedures. (**B1**–**B3**) Isolation of fresh amniotic membrane (fAM) from fetal membrane. (**B4**–**B6**) Preparation of decellularized and lyophilized amniotic membrane (DL-AM). (**B7**–**B12**) Surgical procedures: endometrial injury in rats to induce IUA and DL-AM transplantation. (**B13**) Characterization of fAM vs. DL-AM by gross imaging, light microscopy, SEM, and H&E. (**B14**) Fibrosis evaluation post-surgery (Van Gieson staining at 3, 7, 14, and 28 days shows reduced fibrosis in the DL-AM group). (**B15**) Quantified fibrotic area confirms significant reduction vs. IUA group (* *p* < 0.05), but still higher than control (Copyright: © 2021 Chen et al. (Spandidos), licensed under CC BY-NC-ND 4.0) [103].

The extracellular matrix (ECM) is a highly dynamic and complex network of proteins, glycoproteins, and glycosaminoglycans that provides structural and biochemical support to surrounding cells [99]. Over the past few decades, the understanding and utilization of ECM in tissue engineering and biomedical applications has significantly advanced. One of the key innovations in this field is the extraction and decellularization of the ECM from tissues and organs to create biological scaffolds (Figure 5A) [6]. These scaffolds retain the native ECM architecture, providing a framework conducive to tissue regeneration. Furthermore, the ECM has shown promise as an anti-adhesion barrier in post-surgical applications, preventing the formation of adhesions that can lead to severe complications [100].

The decellularized extracellular matrix (dECM) helps prevent adhesions by acting as a biocompatible physical barrier between healing tissues, minimizing direct contact and reducing fibroblast proliferation [101]. The key structural proteins in the ECM, such as collagen and elastin, are retained in the dECM. Collagen, the most abundant protein, provides tensile strength and structural support, while elastin allows tissues to regain their shape after deformation, imparting elasticity. Additionally, glycoproteins like fibronectin and laminin play crucial roles in cell adhesion, migration, and tissue organization, further promoting controlled tissue regeneration without triggering inflammation. Embedded growth factors within the dECM regulate essential cellular processes, including proliferation, differentiation, and repair, supporting healing [102]. Furthermore, the dECM’s structure can be tailored into films, hydrogels, or sponges (Figure 5A). Its slow degradation provides a sustained barrier during the critical healing phase, reducing adhesion risks. These unique properties make the dECM an effective material for anti-adhesion applications.

Proteoglycans, composed of core proteins and glycosaminoglycans (GAGs), play a role in the ECM’s ability to withstand compressive forces. One particularly important GAG, hyaluronic acid, maintains tissue hydration and facilitates cellular migration, which is essential for tissue repair and regeneration [104]. Together, these components form an intricate and dynamic network that provides both mechanical integrity and bioactivity, supporting tissue function and homeostasis [105].

One of the primary objectives of decellularization is to eliminate nuclear and intracellular materials while preserving the ECM’s structural and functional integrity [99]. Achieving this balance is crucial not only for reducing immune rejection, but also for retaining the biological signals necessary for tissue regeneration (Figure 5A) [22,106]. Importantly, decellularized ECM can be derived from a wide range of tissues and species—both allogeneic and xenogeneic—without requiring species specificity to the recipient. A summary of common donor tissue sources and their applications is presented in Table 2.

Various methods have been developed for decellularization, each with unique advantages and challenges. **Physical methods** include freeze–thaw cycles, supercritical CO_2_, sonication, and mechanical agitation. Freeze–thaw cycles rely on the formation of ice crystals to rupture cell membranes, while mechanical methods such as perfusion decellularization use pressure or agitation to remove cells. These techniques are often less damaging to the ECM, but may not entirely remove cellular debris from dense tissues [112]. **Chemical methods** involve detergents like sodium dodecyl sulfate (SDS) or Triton X-100, which solubilize cell membranes. Acids and alkalis, zwitterionic detergents, and hypotonic and hypertonic solutions can also be used to degrade cellular material. While effective, these chemicals must be carefully balanced to prevent damage to ECM proteins [113]. **Enzymatic methods** use proteolytic enzymes like trypsin, dispase, collagenase, and nucleases to degrade cellular proteins and DNA. These methods are often combined with chemical or physical techniques to ensure complete decellularization. The combination of methods is essential for complex organs such as the liver or heart, where uniform decellularization is critical (Figure 5) [114].

As research in this field advances, the optimization of decellularization techniques and the exploration of new applications for ECM-based materials are expected to drive further innovation in biomedical science. The flexibility of ECM scaffolds, which can be sourced from various tissues and species, allows them to be utilized in a wide range of tissue engineering applications, from organ regeneration to wound healing. Importantly, this versatility also aligns well with the translational goals of anti-adhesion barrier development, where ECM-derived materials offer a biologically active and clinically adaptable platform for reducing postoperative complications. With continued refinement, ECM-based biomaterials hold promise not only in experimental models, but also in future clinical settings (Figure 5A) [115].

A 2021 rodent study transplanted a decellularized, lyophilized human amnion (DL-AM) patch onto a uterine injury (unilateral endometrial scarring) [103]. Rats treated with the DL-AM showed attenuated fibrosis: connective tissue growth factor (CTGF) levels in scarred uteri were significantly lower, and MMP-2 (matrix-degrading enzyme) higher, than in untreated injured uteri. This indicates that the amnion graft partially inhibited fibrous adhesion band formation. However, DL-AM alone did not fully restore normal endometrium—fibrotic markers remained above baseline and there was no significant regeneration of endometrial glands by 4 weeks [103]. A key limitation of the current study lies in the fact that the anti-adhesion effect was not complete, thereby affecting the reliability of the results obtained. Given the insufficient follow-up period and the missing functional fertility data in this study, more research is needed to find the best ways to use dECM to treat women with Asherman’s syndrome (Figure 5B).

A separate study explored the use of a decellularized amniotic membrane, populated with adipose-derived stem cells (ADSCs), as a potential treatment option for uterine adhesions in an experimental rat model. Rats receiving ADSC-seeded amnion grafts showed near-normal uterine recovery: by 14 days post-surgery, endometrial thickness, gland count, and fibrosis scores in the ADSC + AM group approached those of uninjured sham animals and were significantly better than AM alone [116]. Mechanistically, the ADSCs on the scaffold modulated the injury site immune environment—lowering pro-inflammatory TNF-α and IL-1β, and increasing anti-inflammatory factors (bFGF, IL-6)—promoting a regenerative response. Despite improved histology, fertility outcomes remained suboptimal in this model; high miscarriage rates persisted even with ADSC treatment, possibly due to residual uterine receptivity issues [116]. The approach shows promise for restoring uterine function, but larger studies are needed to optimize timing and dosing, as well as evaluate pregnancy success.

#### 2.1.2. Polysaccharides

##### Hyaluronic Acid

Hyaluronic acid (HA), also known as hyaluronan, is a naturally occurring glycosaminoglycan found in the ECM of most connective tissues in the human body. HA’s viscoelasticity and water retention, alongside biocompatibility, biodegradability, and anti-inflammatory properties, make it a leading biomaterial in anti-adhesion barriers. It has been the foundation of many anti-adhesion products, with multiple FDA-approved formulations, making it a gold standard in post-surgical adhesion prevention.

HA modulates macrophage activity, reducing the inflammatory cytokine secretion that leads to adhesion formation. Its non-immunogenic nature and capacity for modifying biological responses have positioned it as an ideal candidate for surgical applications, particularly in the prevention of post-surgical adhesions, including those arising from peritoneal injury [117]. HA also inhibits fibroblast proliferation and fibrin deposition, which are key factors in adhesion development [15]. A well-known example of its clinical use is Seprafilm^®^ (hyaluronic acid/carboxymethylcellulose film), which is FDA-approved and widely used as a physical barrier to reduce intra-abdominal and pelvic adhesions following surgery [118].

**Figure 6:**
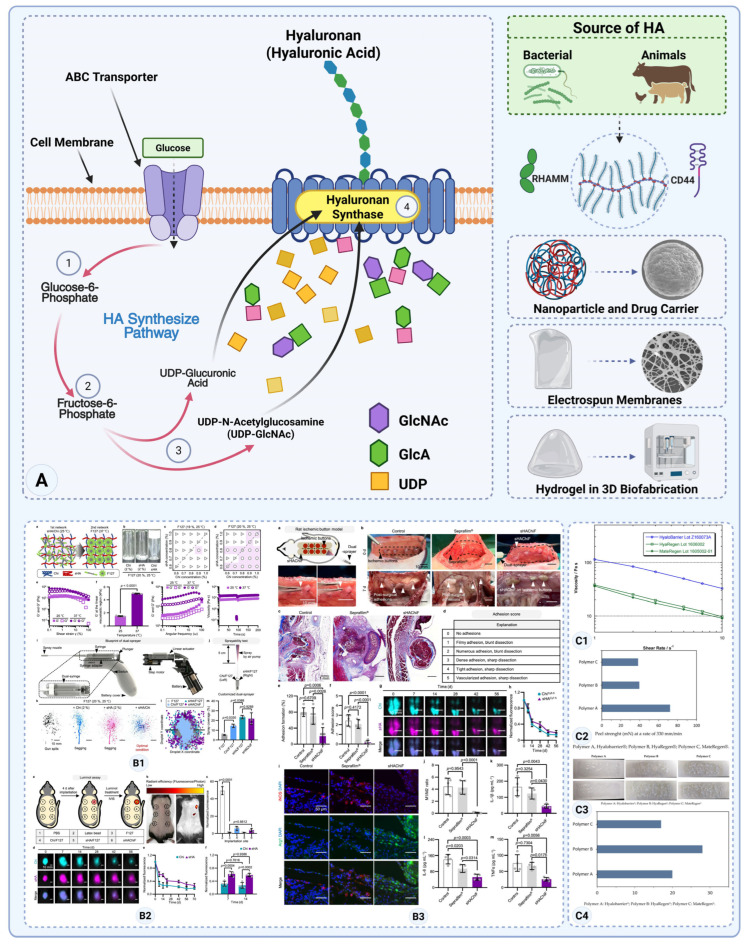
(**A**) Illustration of the hyaluronan (HA) synthesis pathway. Glucose enters the cell via an ABC transporter and is converted to glucose-6-phosphate (1), followed by fructose-6-phosphate (2). Through a series of enzymatic reactions, these intermediates lead to the formation of UDP-glucuronic acid and UDP-N-acetylglucosamine (UDP-GlcNAc) (3). These two precursors are used by the enzyme hyaluronan synthase (4) to polymerize hyaluronan, which is then transported out of the cell. The figure also depicts different sources of HA (bacterial and animal) and its various biomedical applications, including drug carriers, electrospun membranes, and hydrogels in 3D biofabrication (created with Biorender.com). (**B1**) sHAChiF hydrogel showed rapid gelation, strong viscoelasticity, and effective sprayability with uniform coverage using a custom dual-spray system. (**B2**) sHAChiF showed low inflammation (via luminol assay) and sustained in vivo retention of both sHA and chitosan for up to 14 days, confirmed by IVIS imaging and fluorescence quantification. (**B3**) sHAChiF reduced macrophage aggregation and inflammation at injury sites, with strong localization of sHA and chitosan. Flow cytometry showed preferential sHA uptake by small peritoneal macrophages, supporting targeted anti-inflammatory effects (Copyright: © 2024 Song et al. (Springer Nature), licensed under CC BY-NC-ND 4.0) [120]. (**C1**) Hyalobarrier^®^ (Gel A) (Anika Therapeutics, Inc., Bedford, MA, USA) showed significantly higher viscosity across shear rates compared to HyaRegen^®^ (BioRegen Biomedical (Changzhou) Co., Ltd., Changzhou, China) and MateRegen^®^ (BioRegen Biomedical (Changzhou) Co., Ltd., Changzhou, China), indicating superior mechanical stability. (**C2**) Hyalobarrier^®^ exhibited the highest peel strength among the tested HA gels, indicating stronger tissue adhesion than HyaRegen^®^ and MateRegen^®^. (**C3**) Peel strip images showed less residue and tearing with Hyalobarrier^®^, confirming its stronger and more cohesive adhesion compared to HyaRegen^®^ and MateRegen^®^ after repeated tests. (**C4**) HyaRegen^®^ required the highest average extrusion force, while MetaRegen^®^ required the lowest, indicating easier application for MetaRegen^®^ but greater viscosity for HyaRegen^®^ [121].

Hyaluronic acid is a linear polysaccharide composed of repeating disaccharide units of D-glucuronic acid and N-acetyl-D-glucosamine. These disaccharide units are linked by β-1,4 and β-1,3 glycosidic bonds [119]. HA’s molecular weight can vary significantly, ranging from 20 kDa to several million Daltons (Figure 6A) [119]. The molecular weight of HA is key to its function, influencing its rheological properties, water retention capabilities, and biological activity.

In the body, HA interacts with proteins, such as CD44 and Receptor for Hyaluronan Mediated Motility (RHAMM), which play roles in cell signaling, migration, and inflammation (Figure 6) [122,123]. Its highly hydrophilic nature allows it to form viscous gels, making it excellent for peritoneal injury treatments.

HA-based anti-adhesion barriers have become an important strategy to mitigate postoperative adhesions in abdominal and uterine surgeries. Established products like Hyalobarrier (gel) and Seprafilm (sheet) have demonstrated clinical efficacy in reducing adhesion formation—particularly in gynecologic and colorectal surgeries—with acceptable safety profiles [124]. Newer entrants such as thermosensitive gels (Mediclore and analogues) offer easier application and have shown success in trials (especially in pelvic surgeries), though their impact can vary by use case.

A notable example is ABT13107, a thermosensitive HA–poloxamer gel that transitions from liquid to gel at body temperature. In a randomized clinical trial involving 192 women undergoing hysteroscopic surgery, ABT13107 was shown to be non-inferior to Hyalobarrier in reducing intrauterine adhesions, with no serious adverse events reported. Its ease of application and favorable safety profile underscore the potential of next-generation HA barriers to offer both practicality and performance in delicate surgical fields [125].

Similarly, an experimental sprayable dual-network hydrogel composed of sulfated hyaluronic acid (sHA) and chitosan demonstrated superior adhesion prevention to Seprafilm^®^ in a rat ischemic button model. This hydrogel, delivered via a dual-syringe sprayer, forms a conformal gel film upon contact and modulates local inflammation by promoting an anti-inflammatory (M2) macrophage phenotype. In addition to providing effective physical coverage, it actively dampens pro-adhesion immune responses—highlighting a promising direction for HA-based barriers that combine mechanical and immunomodulatory strategies. Though still in preclinical stages, this platform addresses key shortcomings of current barriers in terms of surface coverage, inflammation control, and bioresorption (Figure 6(B1–B3)) [120].

A recent meta-analysis of 12 clinical trials demonstrated that hyaluronic acid (HA) gel application significantly reduces the incidence and severity of postoperative adhesions in gynecological surgery. Patients treated with HA exhibited a notably lower rate of moderate or severe adhesions (*p* = 0.0010) and a significantly smaller proportion of adhesion-affected sites (*p* < 0.00001) compared to controls. These findings highlight HA’s clinical value as an anti-adhesion barrier and support its broader implementation in reproductive surgeries [126].

Chemically modified hyaluronic acid (HA) gels exhibit distinct physical properties that influence their effectiveness as anti-adhesion barriers. A comparative study evaluating three commercial formulations—Hyalobarrier^®^, HyaRegen^®^, and MetaRegen^®^—found that Hyalobarrier^®^ demonstrated significantly higher viscosity (114 Pa·s), elasticity (G’ = 100 Pa), and peel strength (72 mN) compared to the other gels. These properties suggest a stronger capacity to remain in place and resist deformation post-application, potentially enhancing the gel′s anti-adhesion efficacy. However, this came with a moderate increase in extrusion force (20 N), indicating a trade-off between mechanical stability and ease of application. These findings highlight the importance of gel composition in clinical performance (Figure 6(C1–C4)) [121].

##### Carboxymethyl Cellulose (CMC)

**Figure 7:**
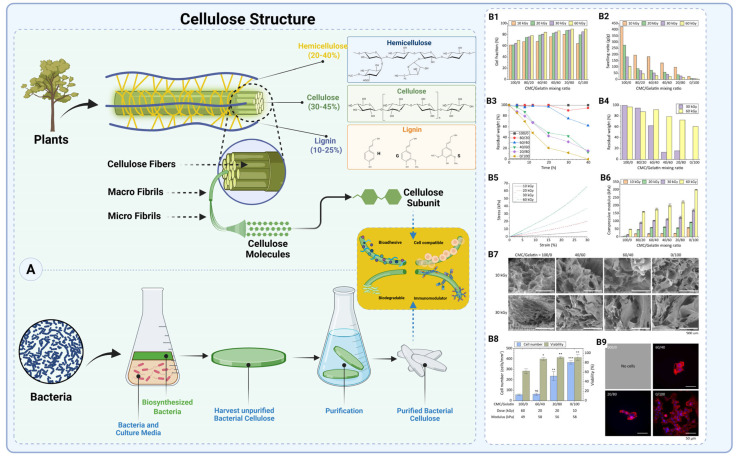
**Structure and sources of cellulose from plants and bacteria**. (**A**) Cellulose exhibits high biocompatibility, bioadhesiveness, biodegradability, and immunomodulatory properties, making it suitable for biomedical applications (created with Biorender.com). (**B1**,**B2**) Gel fraction (**B1**) and swelling ratio (**B2**) of CMC/gelatin hydrogels with varying compositions and radiation doses. B3–B4: In vitro enzymatic degradation of CMC/gelatin hydrogels (**B3**) and residual weight after 40 h in protease at 30 and 60 kGy (**B4**). (**B5**–**B7**) Stress–strain curves (**B5**), compressive moduli (**B6**), and SEM cross-sectional images (**B7**) of hydrogels with varying compositions and irradiation doses. (**B8**–**B9**) Cell viability and number of 3T3-Swiss fibroblasts on hydrogels (**B8**), and representative fluorescence images of remaining adhered cells (**B9**) (reproduced with permission, Elsevier) [141]. The significance levels are indicated as follows: *** for *p* < 0.001, ** for *p* < 0.01, * for *p* < 0.05, and ns for non-significant differences.

Carboxymethyl cellulose (CMC) is derived from cellulose, the most abundant biopolymer on Earth, primarily found in plant cell walls and produced by certain bacteria [127]. Plant-derived cellulose is extracted from wood, cotton, and other fibers, often in combination with hemicellulose, lignin, and pectin [128]. In contrast, bacterial cellulose (BC), synthesized by *Gluconacetobacter xylinus*, features a highly pure nanofibrillar structure, offering high porosity, elasticity, and crystallinity (Figure 7A) [129,130]. This bottom–up biosynthesis process is highly sustainable and cost-effective, often utilizing agricultural waste as a carbon source [119,131]. Structurally, cellulose is composed of *β*(1→4)-linked D-glucose units, with three hydroxyl (-OH) groups per glucose ring. By chemically modifying these hydroxyl groups with carboxymethyl (-CH_2_-COOH) substitutions, cellulose becomes water-soluble CMC, which is more versatile for biomedical applications production [132]. The degree of substitution (DS) and molecular weight are key parameters determining CMC’s solubility, viscosity, and gel-forming ability [132,133,134]. This chemical modification imparts hydrophilicity and biocompatibility, making CMC an excellent anti-adhesion barrier material [9].

CMC absorbs water and swells, forming a hydrogel-like layer that physically separates tissues during healing, preventing direct tissue contact and reducing adhesion formation [135]. Often combined with HA, as in Seprafilm*^®^*, CMC improves mechanical stability and prolongs retention time at the surgical site [136]. These formulations can be processed into films, sponges, or hydrogels, allowing for versatile surgical application [137]. Injectable CMC solutions also enable minimally invasive laparoscopic delivery [138]. Furthermore, CMC degrades naturally into non-toxic byproducts; however, its rapid degradation and tendency for excessive swelling can limit barrier effectiveness [9]. Crosslinking strategies have been used to mitigate these issues by extending retention and reducing hydrogel expansion [139]. Despite its advantages, CMC has limitations. CMC films and hydrogels can be fragile, posing handling difficulties during surgery [140]. Additionally, uncrosslinked CMC may degrade too quickly, compromising its function as a sustained barrier. In some cases, excessive swelling of CMC-based hydrogels may exert pressure on adjacent tissues, potentially affecting healing and even contributing to adhesion formation rather than preventing it. If not fully absorbed, residual CMC may interfere with tissue remodeling [141].

A comparative summary of the most widely used commercial CMC-based anti-adhesion barriers, including their formulations, surgical applications, advantages, and limitations, is presented in Table 3 to provide a clear overview of their clinical utility.

**CMC–Gelatin Barrier:** A recent study introduced a new approach for designing anti-adhesion barriers by combining carboxymethyl cellulose (CMC) and gelatin into a hybrid hydrogel crosslinked via γ-radiation. This formulation, particularly at a 60:40 CMC-to-gelatin ratio, achieved compressive moduli between 20 and 100 kPa, closely mimicking abdominal tissue mechanics. The CMC component effectively reduced fibroblast adhesion, while gelatin supported the viability of limited attached cells. Moreover, enzymatic degradation tests confirmed that gelatin content enabled controlled biodegradation, modulated by radiation dose. These hydrogels also showed reduced foreign body response and strong anti-adhesive properties, highlighting their potential as biocompatible, tunable barrier materials for postoperative adhesion prevention (Figure 7B) [141].

**CMC/Pullulan Composite Barrier:** Researchers have developed drug-free composite barriers of CMC and pullulan (a polysaccharide) in various physical forms. Researchers created three formulations: a solution, a flexible film, and a thermo-sensitive gel from CMC + pullulan (with small amounts of Pluronic in some mixtures) [142]. These materials are biodegradable over ~30 days and were designed to promote tissue healing while blocking adhesions. In a rat laparotomy model, the CMC–pullulan barriers showed excellent biocompatibility (no toxicity or inflammation) and impressive efficacy. They were compared against an existing Korean adhesion barrier called Medicurtain (an anti-adhesion gel already on the market). Medicurtain prevented postoperative adhesions in ~42% of animals, whereas the new CMC–pullulan formulations prevented adhesions in 100% of the treated rats. In other words, all rats treated with optimized CMC–pullulan barriers had a completely adhesion-free recovery, a dramatic improvement over the control agent. The study also noted enhanced tissue regeneration at the surgical site with these formulations, suggesting that the materials might actively support healing [142].

These results are very promising, though translation to humans is pending. The formulations will need further testing in large animals and eventual clinical trials. Also, achieving 0% adhesions in humans is unlikely, but even a significant relative reduction would be valuable. The absence of any drug (the barrier’s effect is purely mechanical/biological) means fewer regulatory hurdles and possibly fewer side effects if brought to market.

Nonetheless, with an optimized degree of substitution and molecular weight, CMC remains a highly effective biomaterial in tissue engineering and adhesion prevention. Sodium carboxymethyl cellulose (Na-CMC), in which sodium ions neutralize carboxyl groups, enhances water solubility for applications requiring rapid dissolution and uniform distribution [143]. These features make CMC a versatile and clinically relevant material, particularly in anti-adhesion barriers and regenerative medicine.

##### Chitosan

After cellulose, chitosan is the second most abundant biopolymer found in living organisms [144]. Chitosan is non-toxic, biodegradable, and exhibits minimal immune response when used in vivo. Its degradation products—primarily glucosamine and acetylglucosamine—are naturally occurring metabolites that the body can absorb and eliminate. Chitosan can support cell adhesion, proliferation, and differentiation, which are key characteristics required for tissue engineering scaffolds. Additionally, its cationic nature promotes wound healing by facilitating the migration of fibroblasts and keratinocytes, which are crucial for tissue regeneration [145].

**Figure 8:**
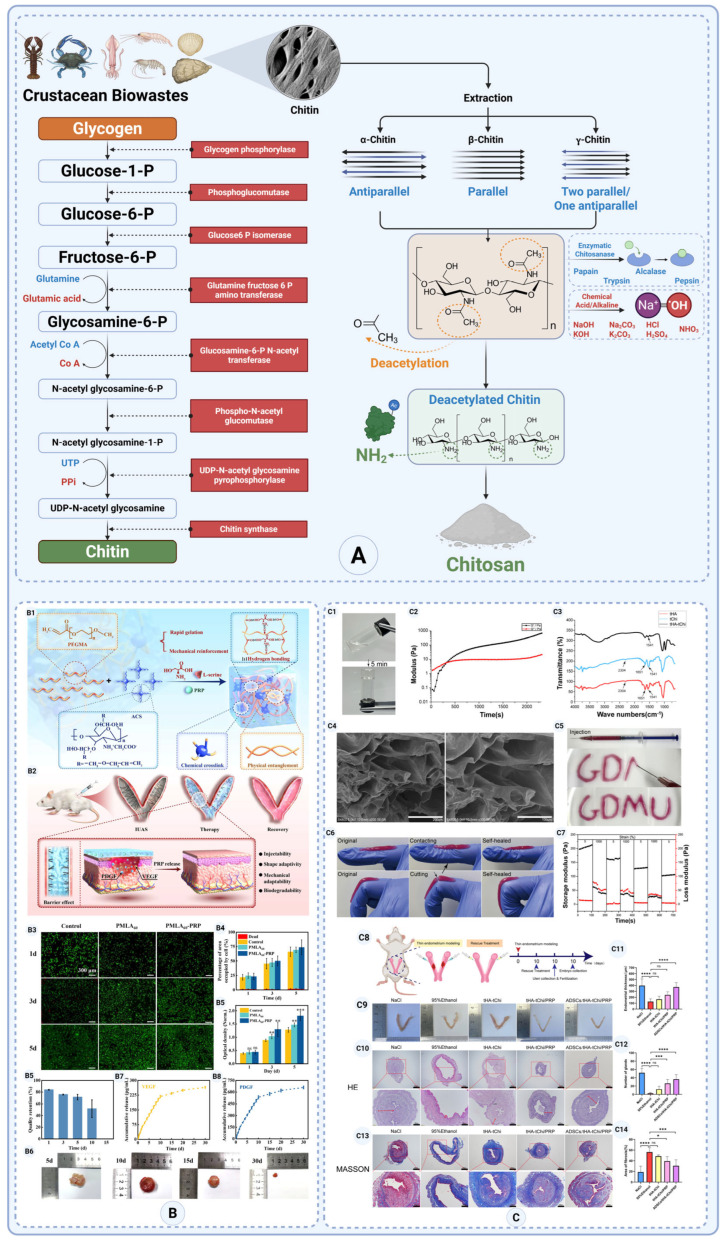
(**A**) Synthesis of the pathways of chitin in crustacean biowastes and extracted chitosan (created with Biorender.com). (**B1**,**B2**) Design of PEGMA/L-serine/PRP hydrogel and its anti-adhesion and PRP-release function. (**B3**–**B5**) Cell viability, live/dead staining, and CCK-8 assay confirm good biocompatibility. (**B6**–**B8**) Hydrogel shows controlled degradation, sustained VEGF/PDGF release, and in vivo degradation confirmed over 30 days (reproduced with permission, Elsevier) [150]. (**C1**–**C7**) tHA-tChi hydrogel shows sol–gel transition, injectability, self-healing, and stable rheology. (**C8**–**C14**) In vivo, it improves endometrial thickness, gland number, and reduces fibrosis (reproduced with permission, Elsevier) [151]. The significance levels are indicated as follows: **** *p* < 0.0001, *** for *p* < 0.001, ** for *p* < 0.01, * for *p* < 0.05, and ns for non-significant differences.

From a structural point of view, both chitin and cellulose are polymers of monosaccharide made up of β-(1→4)-2-acetamido-2-deoxy-β-D-glucopyranose and β-(1→4)-2-deoxy-β-D-glucopyranose units, respectively. Chitin is generally represented as a linear long-chain homo-polymer composed of N-acetyl glucosamine units [poly(N-acetyl-β-D-glucosamine)] [146,147]. It is obtained by deacetylating chitin, resulting in a linear polysaccharide composed of glucosamine and N-acetylglucosamine units. The degree of deacetylation (DD) determines the proportion of free amine groups (-NH_2_) in the structure, which influences its solubility and reactivity (Figure 8A) [148]. This structure provides chitosan with a cationic nature, making it unique among polysaccharides and capable of interacting with negatively charged biological molecules such as proteins and cell membranes, which makes it a favorable biopolymer for tissue engineering applications [144]. However, practical limitations include variability in molecular weight and degree of deacetylation due to its natural sourcing from shellfish, as well as regulatory hurdles. Although chitosan is classified as Generally Recognized as Safe (GRAS) by the U.S. FDA for certain food applications, its medical-grade use—especially in injectable or implantable products—requires rigorous purification to meet endotoxin, sterility, and biocompatibility standards, which can complicate its clinical translation [149].

Chitosan exists in different structural forms, known as alpha (α-), beta (β-), and gamma (γ-), presenting unique properties that influence its suitability for anti-adhesion barrier applications [152,153]. These variants influence the polymer’s solubility, crystallinity, and overall functional properties.

**α-Chitosan**, derived from crustacean shells, has an anti-parallel polymer arrangement, resulting in high crystallinity and mechanical strength [154,155]. While its low solubility limits its direct use as an injectable or hydrogel-based barrier, crosslinked α-chitosan films can provide durable physical barriers that prevent tissue adhesion [156]. Its biocompatibility makes it ideal for long-term anti-adhesion scaffolds in tissue repair (Figure 8) [157,158].

**β-Chitosan**, found in squid pens, has a parallel chain structure, leading to lower crystallinity, higher solubility, and flexibility (Figure 8) [157,159]. This makes β-chitosan hydrogels and coatings highly effective as injectable anti-adhesion materials, particularly for laparoscopic surgeries [160,161]. Its swelling and higher water absorption helps maintain a hydrated environment, reducing fibrin bridge formation, a key factor in adhesion development [162,163].

**γ-Chitosan**, with mixed-chain alignments, balances solubility and mechanical properties, making it an optimal candidate for bioadhesive films and hydrogels (Figure 8) [159]. It has better flexibility than α-chitosan but is less soluble than β-chitosan [164]. γ-chitosan is known for having enhanced water solubility while maintaining reasonable mechanical properties [158]. It also offers a sustained anti-adhesion effect while allowing controlled degradation to support wound healing and tissue regeneration [165].

By selecting the appropriate chitosan variant, anti-adhesion barriers can be tailored for specific surgical applications, optimizing efficacy, durability, and biocompatibility in adhesion prevention. Table 4 gives a summary of the differences between these three chitosan groups.

To design effective anti-adhesion barriers, chitosan’s properties can be tailored by adjusting its degree of deacetylation, molecular weight, and chemical modifications like crosslinking [166]. Its moderate mechanical strength ensures flexibility and controlled degradation, essential for adhesion prevention. Blending chitosan with polymers (e.g., collagen, alginate) enhances its strength for barrier durability [167]. Chitosan’s antibacterial, hemostatic, and exudate-absorbing properties support wound healing, reducing infection risk [168]. It promotes angiogenesis, granulation tissue formation, and re-epithelialization, aiding tissue recovery [169,170]. Its versatility in forming hydrogels, films, or nanofibers enables moisture retention and controlled drug release, making it ideal for anti-adhesion and regenerative applications.

Recently, researchers developed an injectable, in situ gelling hydrogel using allyl-functionalized chitosan (a chemically modified chitosan) combined with L-serine as a trigger and loaded with platelet-rich plasma. This hydrogel is mechanically adaptive, conforming to the uterus shape, and degrades over time [150]. In a rat model of intrauterine injury/adhesions, the chitosan–PRP hydrogel markedly reduced post-surgical adhesions and fibrosis. Treated rats had significantly less collagen scar deposition and increased endometrial cell proliferation, leading to near-normal endometrial architecture. Importantly, fertility was restored in treated rats (evidenced by successful pregnancies in the IUA model), whereas untreated rats remained infertile. No notable toxicity or inflammation was observed, indicating good biocompatibility (Figure 8B) [150]. This hydrogel serves as an excellent example of a material that could be developed to prevent adhesions following abdominal surgeries.

Another novel formulation is a chitosan–lignosulfonate/poloxamer 127 thermosensitive hydrogel, also loaded with PRP growth factors [171]. This gel remains liquid at room temperature but solidifies in the body. In a recent study, it was injected into a rat uterine injury model after creating adhesions. After 2 weeks, rats treated with the chitosan composite hydrogel showed an almost normal uterine cavity with regenerated endometrium, in contrast to untreated rats that had obliterated cavities and necrotic tissue. Histologically, the hydrogel group had dramatically reduced fibrosis: collagen deposition in the uterine wall was ~29% vs. ~50% in untreated controls. The hydrogel also promoted angiogenesis in the endometrium, evidenced by CD31+ blood vessels being restored to near-normal levels. These outcomes indicate enhanced endometrial repair and a strong anti-adhesion effect. The material proved safe in vivo, with no significant inflammatory reaction, and is fully biodegradable [171]. To address the issue of intrauterine adhesion (IUA), a new injectable hydrogel was created using thiolated chitosan (tChi) and thiolated hyaluronic acid (tHA); this hydrogel is thermosensitive, meaning its properties change with temperature, and its development is documented in reference [151]. This disulfide-crosslinked system exhibited favorable properties, including rapid sol–gel transition, self-healing, and excellent injectability. In vivo imaging confirmed prolonged retention of the hydrogel components in the uterine cavity for up to 14 days. Functionally, the hydrogel demonstrated anti-inflammatory effects by reducing macrophage aggregation and lowering inflammatory marker expression in a hepatic injury model. In a rat model of IUA, hydrogel-treated animals showed significantly improved endometrial regeneration, with greater endometrial thickness, increased gland numbers, and reduced fibrosis compared to untreated controls (*p* < 0.01). These results highlight the hydrogel’s dual role as both a physical barrier and an active modulator of inflammation, offering a promising strategy for effective adhesion prevention (Figure 8C) [151].

##### Alginate

Alginate has been widely explored in biomedical applications due to its excellent biocompatibility, non-toxicity, and ability to form hydrogels. Its use has expanded into various fields, including wound healing, drug delivery, and tissue engineering [22]. In the context of anti-adhesion barriers, alginate-based hydrogels have been investigated for their potential to prevent post-surgical adhesions by creating a temporary physical barrier that separates injured tissues during the healing process [172,173]. However, despite its promising properties, alginate alone is not recommended for use in anti-adhesion applications due to its lack of bioactivity, weak mechanical stability, and rapid degradation in physiological environments [174]. To enhance its efficacy, alginate is typically blended with bioactive biomaterials that improve adhesion resistance, mechanical durability, and regenerative potential [175,176]. Several studies have demonstrated that incorporating collagen, chitosan, gelatin, or hyaluronic acid into alginate hydrogels significantly improves their anti-adhesion performance [177]. For example, alginate–collagen composites provide enhanced bioactivity, mimicking the extracellular matrix while promoting cellular interactions and tissue integration [178]. Similarly, alginate–chitosan hybrids improve mechanical stability and prolong degradation, ensuring the barrier remains intact for an extended period [179]. Functionalization with bioactive peptides, such as Arg-Gly-Asp (RGD) motifs, has also been explored to further optimize these materials for clinical use, as has the incorporation of anti-inflammatory agents and growth factors [180].

**Figure 9:**
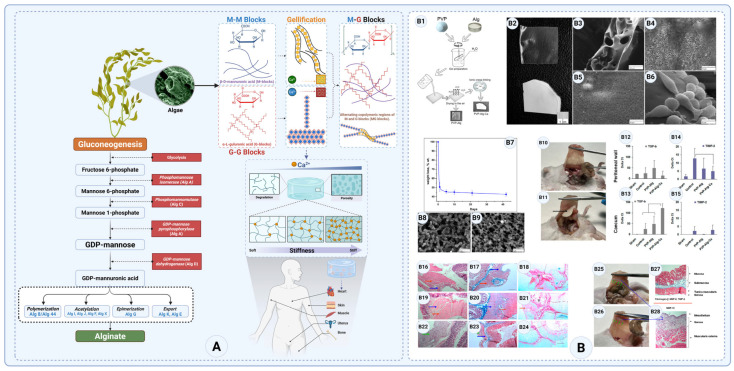
(**A**) Biosynthesis pathway of alginate, structure and ionotropic gelation mechanism of alginate, and applications in tissue engineering (created with Biorender.com). (**B1**) Preparation schematic of PVP-Alg and PVP-Alg-Ca films. (**B2**–**B6**) Film morphology and SEM: porous vs. smooth surfaces and cell adhesion. (**B7**–**B9**) Dissolution behavior and surface changes over time. (**B10**–**B15**) Surgery site views and gene expression (TGF-β, TIMP-2); *p* < 0.05. (**B16**–**B24**) Histology of injured sites (H&E, Masson, Sirius Red) showing tissue structure. (**B25**–**B28**) Adhesion formation and histological origin in treatment groups (Copyright: © 2023 (Copyright: © 2023 Mao et al. (Frontiers), licensed under CC BY 4.0 et al. (MDPI—*Materials*), licensed under CC BY 4.0)) [191].

Alginate is a linear polysaccharide composed of β-D-mannuronic acid (M-blocks) and α-L-guluronic acid (G-blocks), capable of forming hydrogels via ionic crosslinking with divalent cations like calcium (Ca^2+^), making it suitable for biomedical applications (Figure 9A) [22,181]. The relative ratio of M and G blocks significantly affects gel strength, degradation rate, and hydrogel stability—key considerations in anti-adhesion barrier design [182]. Bacterial-derived alginates, such as those produced by *Pseudomonas aeruginosa* and *Azotobacter vinelandii*, typically have higher M-block content, resulting in softer, more elastic gels that degrade quickly and may lack sufficient mechanical strength for long-term barrier use [183,184]. In contrast, high-G alginates form stiffer, more stable hydrogels due to enhanced ionic crosslinking, making them better suited for prolonged adhesion prevention [153,154]. Fine-tuning the M/G ratio enables the customization of mechanical properties and degradation profiles to match surgical site requirements and improve clinical outcomes.

The structural properties of alginate play a crucial role in designing effective hydrogels and composite materials for anti-adhesion barrier applications. As a linear polysaccharide composed of β-D-mannuronic acid (M-blocks) and α-L-guluronic acid (G-blocks), alginate’s ability to form hydrogels through ionic crosslinking with divalent cations like calcium (Ca^2+^) makes it a promising material for biomedical applications (Figure 9A). However, the proportion of M and G blocks significantly influences the mechanical properties, degradation rate, and stability of the resulting hydrogel, which are critical factors in anti-adhesion barrier design. Bacteria like *Pseudomonas aeruginosa* and *Azotobacter vinelandii* also produce alginate, primarily forming a biofilm. While the structure is similar, bacterial alginate is softer, with a higher M-block content [184]. This distinction is important when considering alginate sources for biomedical applications, as bacterial-derived alginate may not provide the same mechanical strength required for durable anti-adhesion barriers.

High-G alginates form stronger, more rigid gels due to enhanced ionic crosslinking, making them suitable for applications requiring prolonged barrier function [185]. These hydrogels provide a more mechanically stable structure, ensuring that the barrier remains intact at the surgical site for an extended period, effectively preventing tissue adhesion. In contrast, high-M alginates yield softer, more elastic gels that degrade more rapidly, making them more adaptable to dynamic tissue environments but less suitable for long-term adhesion prevention [186]. By adjusting the ratio of M and G blocks, researchers can fine-tune the hydrogel’s mechanical properties to balance stability and degradation, ensuring optimal performance in clinical settings.

Crosslinking strategies also play a pivotal role in optimizing alginate-based hydrogels for anti-adhesion barriers. While ionic crosslinking with Ca^2+^ allows for rapid gelation and easy hydrogel formation, incorporating multivalent ions such as Ba^2+^ or Sr^2+^ can enhance network stability, prolonging the material’s functional lifespan (Figure 9A) [187,188,189]. Furthermore, dual crosslinking approaches that combine ionic and covalent crosslinking can provide additional mechanical strength and controlled degradation, ensuring that the barrier remains effective during the critical phases of tissue healing [190].

One study evaluated a composite barrier film of soluble sodium alginate and polyvinylpyrrolidone (PVP) versus a calcium-crosslinked alginate film [191]. After creating standardized peritoneal injuries in mice, the soluble PVP–Alg film completely prevented adhesions: no macroscopic adhesions formed and the gene expression of pro-adhesion markers remained at baseline levels, similar to uninjured controls. In contrast, the Ca-crosslinked alginate film (not readily biodegradable) induced a robust foreign-body response—adhesion bands scored as severe (grade 5) and a 5–10× upregulation of adhesion-related genes occurred. These results underscore that a non-crosslinked, fast-dissolving alginate barrier is effective, whereas a persistent alginate scaffold can trigger inflammation and adhesion formation (Figure 9B) [191]. One limitation of this study is that this experiment was short term (7 days) in a murine model. While the study clearly demonstrates the importance of polymer solubility, further studies should confirm that the PVP–alginate film provides durable adhesion prevention over longer periods and does not impair healing.

### 2.2. Synthetic Polymers

#### 2.2.1. Biodegradable Polymers

##### Polycaprolactone (PCL)

Polycaprolactone (PCL) has emerged as a promising synthetic biomaterial for postoperative anti-adhesion barrier films due to its biodegradability, mechanical flexibility, and controlled degradation rate [192]. PCL-based films effectively serve as physical barriers, preventing the adhesion of healing tissues and reducing the risk of fibrotic scarring following surgical procedures [193].

**Figure 10:**
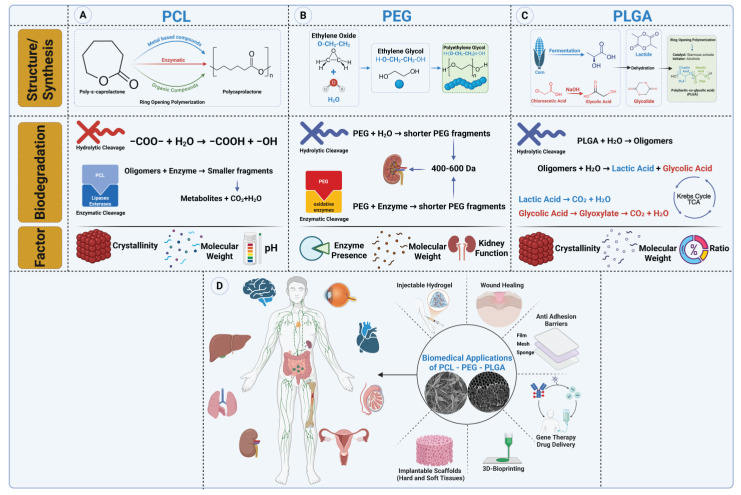
Structure and synthesis pathways of (**A**) PCL, (**B**) PEG, and (**C**) PLGA synthetic biopolymers commonly used in tissue engineering, along with their degradation mechanisms. Key factors influencing the rate and process of degradation, such as molecular weight. (**D**) represents the techniques and biomedical applications of PCL-PEG, and PLGA in tissue engineering (created with Biorender.com).

Structurally, PCL is semicrystalline with a repeating unit of (C_6_H_10_O_2_), characterized by long polymer chains with ester linkages (Figure 10A). Its structure includes five methylene groups between the ester linkages, making it more hydrophobic compared to other polyesters like polylactic acid (PLA) and polyglycolic acid (PGA) [194]. One of PCL’s primary advantages in adhesion prevention is its slow degradation rate, which ensures that the barrier remains intact for an extended period, allowing tissues to heal separately before resorption occurs [195]. Unlike rapidly degrading polymers that break down too soon—potentially exposing healing tissues to adhesion risk—PCL maintains structural integrity for weeks to months, making it highly suitable for adhesion-prevention applications [196]. Its hydrophobic nature also plays a key role in reducing excessive protein adsorption, which can contribute to adhesion formation.

PCL’s long degradation time—often lasting several months to years—can pose limitations for POA prevention, where the ideal barrier lifespan is typically a few weeks to months [197]. Slow degradation can result in chronic foreign body reactions or encapsulation, which may compromise tissue healing or trigger fibrotic responses. To address this, PCL is often blended with polymers such as PLA or PEG to not only accelerate degradation, but also improve cytocompatibility [198]. Blending PCL with hydrophilic or bioactive polymers reduces surface hydrophobicity and enhances wettability, leading to better cell adhesion and viability. For example, PEG-functionalized lactide–caprolactone copolymers demonstrated improved endothelial cytocompatibility and hemocompatibility in vitro [199]. Similarly, PLA–PCL nanofibrous blends have shown enhanced support for fibroblast proliferation and the osteogenic differentiation of stem cells, confirming the favorable cell–material interactions resulting from such modifications [200].

In addition to its mechanical durability, PCL’s customizability makes it adaptable for various surgical applications. By modifying molecular weight or incorporating bioactive coatings, PCL films can be tailored to optimize their barrier properties while ensuring biocompatibility [195]. Surface modifications such as plasma treatment or blending with hydrophilic polymers further enhance its anti-adhesion efficiency by fine-tuning its tissue interaction and degradation profile (Table 5) [201].

Overall, PCL stands out as an effective, long-lasting synthetic biomaterial for postoperative anti-adhesion barrier films, providing sustained protection against tissue adhesions while degrading in a controlled manner. Its combination of mechanical resilience, biocompatibility, and tunable degradation makes it an ideal candidate for clinical use in surgical adhesion prevention.

##### Polyethylene Glycol (PEG)

Polyethylene glycol (PEG) has established itself as a versatile and biocompatible synthetic biomaterial in postoperative anti-adhesion barrier films, primarily due to its hydrophilic nature, low protein adsorption, and tunable degradation properties [207,208]. PEG-based barriers effectively prevent fibrotic adhesion formation by creating a hydrated, non-fouling surface that inhibits cellular attachment and extracellular matrix deposition following surgical procedures [209,210]. Unlike hydrophobic polymers, which may unintentionally promote localized inflammatory responses, PEG remains biologically passive, ensuring minimal immune activation [211]. Furthermore, PEG can be engineered into hydrogels, forming a soft, flexible, and water-retentive barrier that closely mimics natural tissue environments, helping to maintain tissue separation during critical healing phases [212].

PEG’s chemical structure, consisting of repeating ethylene oxide (-O-CH_2_-CH_2_-) units, plays a crucial role in designing anti-adhesion films and hydrogels (Figure 10B) [213]. This highly flexible and hydrophilic backbone allows for the formation of crosslinked networks that retain large amounts of water, making PEG an ideal candidate for hydrogel-based adhesion barriers [214]. In hydrogel formulations, PEG can be chemically crosslinked to control its pore size, mechanical strength, and degradation rate, ensuring that it remains intact long enough to prevent adhesions while gradually dissolving as the tissue heals. Additionally, the ability to adjust molecular weight and branching (linear vs. multi-arm PEG structures) provides further flexibility in designing tailored film or hydrogel formulations with specific mechanical and bioresorption properties [215].

For film-based barriers, PEG’s inert and elastic properties allow for the creation of thin, flexible sheets that can conform to tissue surfaces while maintaining a hydrated, non-adhesive interface [216]. Unlike rigid or brittle polymers, PEG-based films maintain structural integrity without causing additional mechanical irritation, making them highly suitable for minimally invasive adhesion prevention strategies [217].

While PEG is highly effective at minimizing adhesion formation, its rapid degradation profile presents a potential limitation. Native PEG undergoes both hydrolytic and oxidative degradation, often leading to early dissolution before the adhesion risk subsides [218]. In particular, oxidative degradation—driven by reactive oxygen species (ROS) in inflamed tissues—can compromise PEG’s long-term stability in vivo, reducing its effectiveness in maintaining prolonged tissue separation [219]. This necessitates crosslinking strategies or blending with more stable polymers to extend its functional duration. In response, PEG derivatives, such as PEG–diacrylate or PEG–polyesters, have been developed to enhance mechanical stability and control degradation rates for optimized anti-adhesion applications [220,221,222]. These modifications aim to maintain the efficacy of PEG-based anti-adhesion barriers during the critical healing period.

In addition to tunable degradation, PEG’s mechanical properties can be tailored by adjusting molecular weight or crosslinking density, allowing for precise control over barrier durability and elasticity. Advanced thermoresponsive PEG hydrogels have also emerged as promising injectable formulations, enabling minimally invasive deployment while providing localized adhesion prevention [223].

Overall, PEG is a highly effective biomaterial for anti-adhesion barrier films, offering exceptional biocompatibility, non-fouling surface properties, and adaptability in formulation. However, optimizing its degradation rate remains a key challenge, as excessively rapid resorption may compromise long-term efficacy.

##### Poly Lactic-Co-Glycolic Acid (PLGA)

Poly(lactic-co-glycolic acid) (PLGA) has been widely utilized in biomedical and pharmaceutical applications due to its biodegradability, biocompatibility, and tunable degradation rate [224]. Its application in postoperative anti-adhesion barrier films is particularly valuable as it provides a temporary protective layer that prevents tissue adherence during healing [225]. PLGA’s ability to degrade into lactic and glycolic acid, which are naturally metabolized by the body, makes it a safe and effective material for adhesion prevention. One of the key advantages of PLGA is its adjustable degradation profile, which is controlled by the ratio of lactic acid (LA) to glycolic acid (GA) [226]. A 50:50 ratio results in a faster-degrading, more amorphous structure, suitable for short-term barrier applications, while a higher lactic acid content (e.g., 85:15 LA:GA) increases crystallinity and hydrophobicity, leading to slower degradation and prolonged tissue separation [226,227,228]. This tunability allows PLGA-based films to be customized for specific surgical applications, ensuring that the barrier remains in place for the necessary duration before being safely resorbed.

The structural properties of PLGA play a crucial role in designing effective anti-adhesion films and hydrogels. Its ester bond linkages enable controlled hydrolysis, while its mechanical flexibility allows for the fabrication of thin, conformable films that adhere to tissue surfaces without causing irritation or excessive mechanical stress (Figure 10C) [229]. Additionally, PLGA can be formulated into injectable hydrogels, which offer minimally invasive application and localized adhesion prevention, particularly in complex surgical sites [230]. However, despite these advantages, rapid hydrolysis in highly aqueous environments can sometimes cause premature degradation, reducing the effectiveness of the barrier before the risk of adhesion formation has fully subsided [231]. PLGA polymers degrade via the bulk hydrolysis of ester bonds and break down into their constituent monomers, lactic acids, and glycolic acids. The hydrolytic cleavage involves reaction with one molecule of water to form acidic and alcoholic end groups. These monomers are naturally eliminated: glycolic acid is excreted renally, while lactic acid enters the Krebs cycle and is metabolized to carbon dioxide and water [232].

To address early degradation concerns, researchers have explored polymer blending (e.g., with PEG or PCL), surface modifications, and controlled crosslinking techniques to extend degradation time and enhance film stability [233,234].

Another limitation of PLGA is its acidic degradation byproducts, particularly in formulations with higher glycolic acid content, which can lead to localized pH drops, potentially triggering inflammatory responses and affecting tissue healing [235]. To mitigate this, buffering agents and composite formulations incorporating neutralizing polymers have been investigated to improve biocompatibility and stability in adhesion-prevention applications [236]. Despite these challenges, PLGA remains a cornerstone biomaterial for postoperative adhesion barriers, offering customizable degradation rates, excellent biocompatibility, and adaptable mechanical properties. With ongoing advancements in polymer engineering and composite formulations, PLGA-based anti-adhesion films and hydrogels continue to be optimized for enhanced performance and improved patient outcomes.

#### 2.2.2. Non-Degradable Polymers

##### Expanded Polytetrafluoroethylene (ePTFE)

Expanded polytetrafluoroethylene (ePTFE) derived from polytetrafluoroethylene (PTFE) is a widely used material in tissue engineering and anti-adhesion applications due to its exceptional biocompatibility, chemical inertness, and non-degradable properties [237]. ePTFE is manufactured through a specialized process that expands the material, resulting in a microporous structure [238]. This structure enhances its mechanical properties, such as flexibility and strength, while maintaining the hydrophobic and chemically inert characteristics of PTFE. These features make it an excellent choice for long-term implants in medical applications [239].

In surgical settings, ePTFE is particularly valuable as an anti-adhesion barrier. Its non-reactive nature minimizes the risk of eliciting an inflammatory response or fibrotic encapsulation, common concerns with many implantable materials [240]. The material’s microporous structure allows selective tissue integration while preventing excessive tissue adhesion, making it suitable for use in abdominal and pelvic surgeries. For instance, in hernia repairs, ePTFE serves as a physical barrier that prevents post-surgical adhesions between organs and surrounding tissues, thereby reducing complications such as bowel obstruction or chronic pain [241].

##### Polypropylene (PP)

Polypropylene (PP) is another widely used polymer in tissue engineering, particularly in the fabrication of surgical meshes for hernia repair, pelvic organ prolapse, and other soft tissue reinforcement procedures [242]. Its popularity stems from its excellent mechanical properties, such as high tensile strength and durability. PP’s inertness and non-degradability ensure that it maintains its mechanical integrity over the long term, which is critical for applications requiring permanent structural support [243].

In anti-adhesion applications, PP is often used in composite meshes where one side provides structural support, while the other side, typically coated with a biodegradable or bio-inert material, prevents tissue adhesion [244]. For example, PP meshes used in hernia repairs are sometimes coated with oxidized regenerated cellulose or polylactic acid (PLA) to prevent contact with delicate organs like the intestines, thereby reducing the risk of post-surgical adhesions [245]. These composite meshes offer a dual-function approach, combining the strength of PP with the anti-adhesion properties of biodegradable coatings.

However, PP’s non-degradable nature can lead to complications, particularly in the long term. While it provides excellent mechanical support, its presence in the body can trigger a chronic inflammatory response, which may lead to mesh encapsulation or fibrosis. Additionally, mesh-related complications, such as erosion or infection, may necessitate surgical removal of the implant [246]. To address these issues, lightweight and large-pore PP meshes have been developed to reduce the overall amount of implanted material, thus lowering the inflammatory response and improving patient outcomes [247]. Recent studies have shown that the surface modification of PP meshes can further reduce postoperative adhesion formation and inflammatory complications [248]. In pelvic and uterine surgeries, patient-specific factors—including prior surgical history, anatomical site, tissue sensitivity, and long-term reproductive goals—must be carefully considered when selecting mesh materials. Accounting for these variables helps minimize risks of chronic inflammation, mesh erosion, or fibrosis, thereby improving long-term surgical outcomes. Notably, the U.S. Food and Drug Administration (FDA) has raised concerns regarding the safety and effectiveness of PP meshes, leading to the reclassification of these devices as high-risk (Class III) and the subsequent withdrawal of certain products from the market [249,250]. These developments underscore the need for continued innovation in mesh design, including surface modifications and degradable coatings, to enhance biocompatibility and reduce long-term complications in pelvic surgeries. Despite the challenges associated with its non-degradable nature, PP remains a promising material in tissue engineering due to its affordability, ease of manufacturing, and long-term durability. Its continued use in anti-adhesion applications is supported by ongoing innovations that seek to mitigate its limitations, particularly in relation to biocompatibility and long-term inflammatory responses.

## 3. Elimination and Excretion Routes of Biomaterials

In biomaterial design, what goes in must safely come out. Post-surgical adhesion barriers are thin films, gels, or membranes placed in the peritoneal cavity to prevent tissues from sticking together during healing. They are typically made of biodegradable biopolymers (natural or synthetic), obviating the need for surgical removal. After fulfilling their role (usually within the first week of healing), these materials break down and are eliminated from the body through various pathways. Understanding how different polymers degrade and are excreted—and how factors like molecular weight and composition influence these processes—is crucial for designing safe and effective anti-adhesion barriers. Improper elimination can lead to foreign body reactions, organ accumulation, or toxicity, whereas optimal elimination ensures the barrier does its job and then leaves no harmful trace.

**Figure 11:**
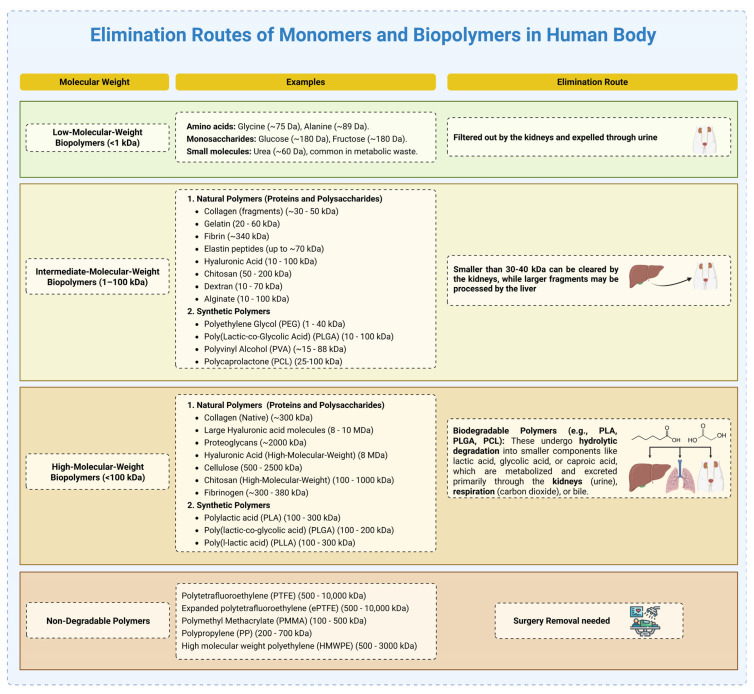
Elimination routes of monomers and biopolymers in the human body [257,258,259,260,261,262,263,264] (created with Biorender.com).

After a barrier degrades in the peritoneal cavity, its remnants are cleared via one or more excretion routes: renal (urinary) excretion, hepatic/biliary excretion, or respiratory excretion (as metabolized CO_2_), sometimes with intermediate steps like lymphatic transport (Figure 11). Also, a summary of key anti-adhesion materials and their primary physiological elimination pathways is provided in Table 6 for clarity.

The predominant pathway depends on the polymer’s molecular size, chemical composition, and breakdown rate:

- **Renal Excretion (Kidney):** The kidneys efficiently filter water-soluble molecules below a certain size. Small polymer fragments, oligomers, or monomers can pass through the glomerular filter and are excreted in urine. For instance, the absorbable HA/CMC barrier Seprafilm breaks down into soluble fragments that are excreted primarily through the kidneys. Radiolabel studies showed that Seprafilm is completely cleared from the body within 28 days, mostly via urine [251]. Similarly, only low-molecular-weight HA fragments (<12 kDa) are able to pass the glomerular barrier—indeed, under normal physiology, only ~1–2% of HA is removed by the kidneys, limited to these small fragments [252]. Hydrophilic synthetic polymers can also be renally eliminated if sufficiently small: e.g., polyethylene glycol (PEG) chains below ~1–40 kDa are largely cleared in urine. In general, polymer degradation products under ~40 kDa (the exact threshold varies) tend toward renal elimination [253].
- **Hepatic and Biliary Excretion (Liver):** Larger polymer fragments that cannot be filtered by the kidney often require clearance via the liver. These may be taken up by hepatic Kupffer cells or by the mononuclear phagocyte system (MPS) in the liver and spleen, then excreted in bile into the feces. Natural macromolecules illustrate this route: the majority of high-molecular-weight HA produced in the body is captured and metabolized by the liver, with the liver sinusoidal endothelium and Kupffer cells clearing most circulating HA each day [252]. Only after enzymatic breakdown into smaller pieces can the metabolites be excreted. Molecular weight is a key factor: for PEG, studies have shown a shift toward hepatobiliary clearance for larger chains—PEG ~50 kDa and above results in increased uptake by Kupffer cells and higher biliary excretion. In fact, very large PEGs (>50 kDa) paradoxically show more liver clearance than mid-sized ones, likely because extremely large polymers are sequestered by phagocytes. Nonetheless, even for quite large polymers (up to ~200 kDa in some cases), urinary excretion can still play a major role [254]. Often, there is a dual pathway: the liver metabolizes or excretes what the kidney cannot, ensuring eventual clearance of the absorbable material.
- **Respiratory Excretion (Metabolic):** Many biodegradable polymers are ultimately broken down into basic metabolites that enter the body’s natural pathways. Polyesters like PLA or PLGA are classic examples—they hydrolyze into lactic acid and glycolic acid. Lactic acid can enter the normal metabolic routes (the citric acid cycle), being converted to CO_2_ and water. Glycolic acid can either be excreted in urine or further metabolized and then eliminated as CO_2_ and water [255]. Thus, a significant portion of PLA/PLGA-based barriers are eliminated via respiration (exhaled CO_2_) and water, rather than as solid polymer remnants. This route is essentially the body “burning off” the polymer fragments as fuel. Other biopolymers that break down into sugars or other metabolic intermediates follow a similar fate—e.g., oxidized cellulose degrades to glucose acid units that can be metabolized to CO_2_ and H_2_O, and collagen/gelatin break into amino acids that are reused or oxidized.
- **Lymphatic Uptake:** Although not an excretion route per se, it is important to note that large polymer fragments in the peritoneal cavity often first enter lymphatic circulation. The peritoneal cavity has lymphatic stomata that drain fluid and large particles to lymph nodes and the thoracic duct. For example, icodextrin (a starch-based polymer used in Adept^®^ adhesion reduction solution) is too large for direct absorption into blood capillaries. Instead, when 4% icodextrin is left in the peritoneum, it is slowly transferred into systemic circulation by peritoneal lymphatic drainage [256]. Once in the bloodstream, enzymes (α-amylase) rapidly depolymerize icodextrin into smaller oligosaccharides, which are then eliminated via the kidneys. In essence, the lymphatics act as a bridge to get large polymers to organs (blood, liver) that can process and excrete them.

The fate of anti-adhesion barrier materials in the body—how they degrade, where their fragments go, and how they exit—is a cornerstone of their design. Molecular weight plays a pivotal role: small breakdown products flow to the urine, very large ones require capture and processing by the liver or immune cells, and many polymer metabolites are simply breathed out as carbon dioxide. Biodegradability and composition determine the speed and nature of this breakdown. A well-designed barrier will maintain an effective presence during the risk period for adhesion formation, then biodegrade into biocompatible units that are efficiently eliminated via renal, hepatic, and metabolic pathways. Understanding these pathways is crucial not only to avoid adverse effects (like organ accumulation, inflammation, or toxicity), but also to comply with safety regulations and optimize patient outcomes. The success of products like Seprafilm^®^, Interceed^®^, and newer hydrogel barriers showcases how leveraging physiological excretion routes can yield materials that are both efficacious in preventing adhesions and safe in the long term. Future developments continue to refine polymer chemistry to further improve these profiles—for instance, by tuning molecular weights or adding enzymatically cleavable linkages—underscoring that in biomaterial design, what goes in must safely come out.

## 4. Common Biopolymeric Adhesion Barriers and Their Degradation

Based on the available information regarding commonly used polymers for postoperative adhesion (POA) prevention, it is evident that both natural and synthetic biopolymers play significant roles in barrier film development.

**Natural biopolymers**—including hyaluronic acid, cellulose derivatives, collagen/gelatin, chitosan, alginate, and starch-based materials—have shown considerable promise. These are traditionally used in the formulation of anti-adhesion barrier films.

**Synthetic polymers**—such as polyesters like polylactic acid (PLA), its copolymer PLGA, polyethylene glycol (PEG)-based hydrogels, and other absorbable polymers—are also widely utilized. These materials are typically formulated into absorbable films or gels designed to be applied over injured peritoneal surfaces to prevent adhesion formation.

The most commonly used commercial adhesion barriers include the following:

- ***Seprafilm^®^ (HA/CMC)***—a film of hyaluronic acid (HA) and carboxymethylcellulose; hydrates into a gel within hours and is fully resorbed in about a week [251].
- ***Interceed^®^ (ORC)***—an oxidized regenerated cellulose fabric; becomes gel-like and is absorbed over ~2–4 weeks, depending on the application site [265].
- ***SprayGel^®^/SprayShield^®^(Radio Systems Corporation, Knoxville, TN, USA) (PEG hydrogel)***—a sprayable PEG-based synthetic gel that polymerizes on tissues and remains for ~5–7 days before degrading and absorbing [266].
- ***Hyaluronic acid gels (*e.g., *auto-crosslinked HA in Hyalobarrier^®^)***—these form a viscous coating that gradually breaks down by enzymatic action over days to weeks depending on the concentration [267].
- ***Polymeric films (PLA/PLGA)***—e.g., polylactide sheets or meshes used experimentally or in combination with meshes; these degrade by hydrolysis over 6-8 weeks, releasing lactic/glycolic acid monomers [268].

Each of these barriers is designed to maintain the separation of tissues for the critical initial healing period (~3–7 days) and then biodegrade. The degradation products—whether they are sugars, acids, or polymer fragments—are then eliminated through the body’s excretory routes.

## 5. Conventional Fabrication Techniques for Anti-Adhesion Barrier Films

Various fabrication techniques have been developed and used to create anti-adhesion barriers or scaffolds from the aforementioned biomaterials. Conventional methods, including gas foaming, freeze drying, porogen leaching, phase separation, and melt molding, are able to create porous structures and thus widely used in developing anti-adhesion barriers. Notably, conventional techniques often have limitations in terms of precise structural control, mechanical tunability, and surface functionalization. To address these limitations, advanced fabrication techniques like electrospinning and bioprinting have recently been developed able to fabricate various customized porous structures with improved controllability and accuracy. Furthermore, research has shown that integrating conventional and advanced fabrication techniques would allow for overcoming the limitations of individual methods, with this approach showing promise for the development of next-generation anti-adhesion barriers with improved performance.

### 5.1. Gas Foaming

**Figure 12:**
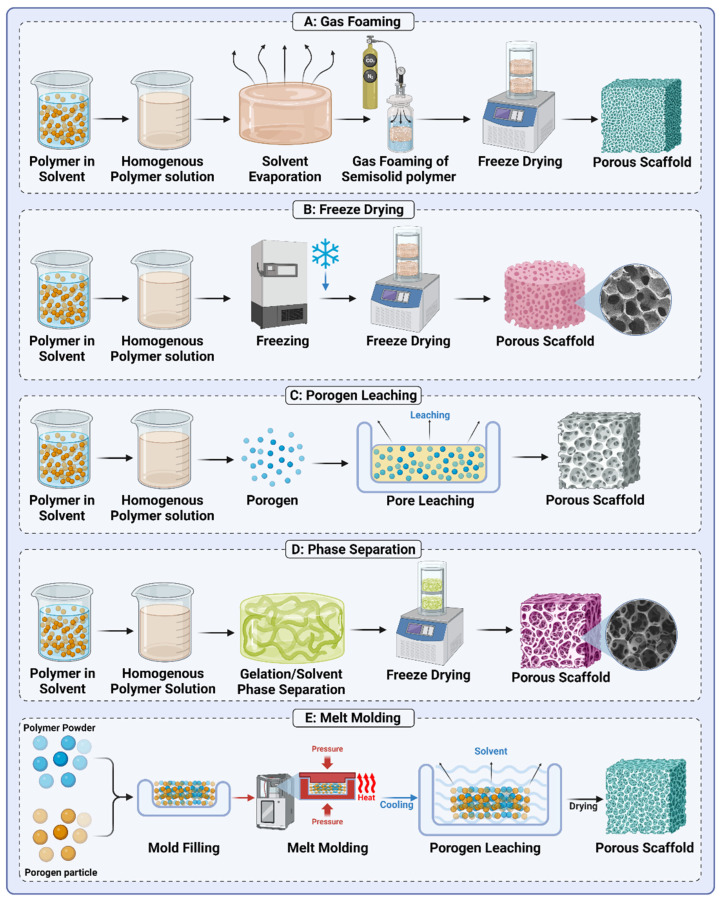
Schematic of conventional scaffold fabrication techniques: (**A**) gas foaming, (**B**) freeze drying, (**C**) porogen leaching, (**D**) phase separation, (**E**) melt molding (created with Biorender.com).

Gas foaming is a technique used in tissue engineering to create porous structures, especially in hydrogels. This method is cost-effective and can achieve high porosity levels up to 90%. It involves the nucleation and growth of gas bubbles within a polymer, which forms an internal phase where porosity is introduced. Importantly, an ideal gas foaming method avoids the use of surfactants and organic solvents, making it suitable for biomedical applications (Figure 12A) [269].

### 5.2. Freeze Drying

Freeze drying, also known as lyophilization, is a technique used in tissue engineering to create scaffolds with specific geometries. It involves three main stages: freezing, primary drying (sublimation), and secondary drying (desorption). This method is particularly useful for generating organ-specific scaffolds that can support tissue development and repair (Figure 12B) [269].

### 5.3. Porogen Leaching

Porogen leaching is a technique used in tissue engineering to create porous polymer scaffolds. It involves incorporating a sacrificial material, such as salt or sugar, into a polymer matrix. After the polymer has set, the sacrificial material is removed, leaving behind a network of pores that enhance cell infiltration and nutrient flow in tissue constructs. This method is effective for mimicking the natural extracellular matrix, promoting better tissue formation (Figure 12C) [269].

### 5.4. Phase Separation

Phase separation is a technique used in tissue engineering to create scaffolds with tailored mechanical properties and controlled pore sizes. This technique in tissue engineering involves several key steps to develop scaffolds with tailored properties. Initially, suitable biodegradable polymers (either natural or synthetic) are selected and dissolved in a solvent to form a homogeneous solution. Phase separation is then induced through methods such as thermal cooling, solvent exchange, or evaporation, causing the polymer to aggregate and separate into distinct phases. As the polymer-rich phase solidifies, it forms a scaffold structure with interconnected pores. After coagulation, the scaffold is washed to remove residual solvents, characterized for its structural and mechanical properties, and then seeded with cells for tissue engineering applications (Figure 12D). This technique enables the precise engineering of scaffolds that support cellular activities, enhancing tissue repair and regeneration [269].

### 5.5. Melt Molding

In the melt molding process, a mixture of polymer powder and a porogen component is placed in a mold and heated beyond the polymer’s glass-transition temperature (Tg). This heating causes the materials to fuse, creating a scaffold that matches the mold’s shape. Afterward, the porogen is removed through leaching, resulting in a porous scaffold (Figure 12E) [270]. Melt molding with porogen leaching is a nonsolvent fabrication method that allows for independent control over the scaffold’s morphology and shape [269]. However, there are some drawbacks, such as the potential presence of residual porogen and high processing temperatures, which limit the incorporation of bioactive molecules.

## 6. Novel Fabrication Techniques for Anti-Adhesion Barrier Films

### 6.1. Electrospinning

Electrospinning uses a high-voltage electric field to draw polymer solutions into ultrafine fibers, which then solidify into a non-woven fabric-like film. This technique can produce nanofibrous mats with high surface area and porosity, suitable as physical barriers. Electrospun anti-adhesion films can be made from biodegradable polymers and even incorporate drugs by techniques like coaxial (core–shell) electrospinning [12,271]. For example, electrospun thermoplastic polyurethane (TPU) nanofiber meshes significantly reduced post-surgical adhesion formation in a rat abdominal adhesion model, with treated animals showing much lower adhesion scores and minimal inflammation [272]. Similarly, an ibuprofen-loaded PEG/silk fibroin core–shell nanofiber membrane, placed between injured peritoneal surfaces, lightened adhesion bands and caused only a low-grade inflammatory reaction in rats [271].

**Advantages:** Electrospinning is relatively simple and allows fine control of fiber diameter and composition; barriers can be imbued with anti-inflammatory or anti-fibrotic agents for added efficacy.

**Limitations:** The resulting films can be mechanically delicate and may require careful handling or fixation. Additionally, achieving uniform thickness and ensuring the barrier stays in place during healing can be challenging in vivo.

### 6.2. Melt Electrowriting

Melt electrowriting (MEW) is an emerging high-resolution additive manufacturing technique that uses an electrostatic field to extrude and precisely deposit microscale polymer fibers from a molten polymer feed. This method creates highly structured porous meshes with controlled fiber alignment and spacing—effectively a microscale 3D-printed fiber scaffold [273]. For anti-adhesion applications, MEW can produce scaffolds that mimic the native architecture of membranes like the peritoneum. A recent study fabricated a “peritoneal scaffold” via melt electrowriting, designing a mesh with fibers intersecting at defined angles (30°, 60°, 90°) to identify an optimal geometry for cell growth [274]. The MEW scaffold with 30° fiber alignment best supported a confluent layer of mesothelial cells—the cells that naturally line the peritoneum—creating a biomimetic barrier. When implanted in a rat abdominal injury model, a mesothelial-cell-seeded MEW mesh acted as a functional anti-adhesion barrier: it physically blocked pro-adhesive cells (like macrophages) from infiltrating the injured site and actively participated in regenerating the damaged peritoneum [274]. The cell-coated MEW film markedly prevented postoperative peritoneal adhesions compared to acellular scaffolds.

**Advantages:** MEW offers exceptional control over scaffold microstructure, enabling tunable pore sizes and alignment to promote healing and integration (for example, by supporting a protective cell layer). The resulting microfiber films are typically stronger and more dimensionally stable than solution-electrospun mats, easing handling and placement.

**Limitations:** MEW is limited to thermally processable polymers (e.g., PCL and PLA) and is a relatively slow fabrication process. The need for seeding patient-derived cells (to fully realize its benefits) adds complexity, and without cells the scaffolds are purely passive barriers. Nonetheless, early in vivo results are encouraging for its translational potential.

### 6.3. Three-Dimensional Bioprinting

Three-dimensional bioprinting (an additive manufacturing technique) builds constructs layer-by-layer using bioinks—typically hydrogels that can include living cells or therapeutic agents. This technique enables custom-shaped barriers and the incorporation of biological components to aid healing [22]. In anti-adhesion applications, 3D-printed hydrogel films have shown promise in both pelvic and general surgery models. For instance, a composite hydrogel of gelatin methacrylate (GelMA) and collagen methacrylate was bioprinted with encapsulated human amniotic mesenchymal stem cells (hAMSCs) and crosslinked by blue light. In a rat model of intrauterine adhesions, this cell-laden printed hydrogel successfully prevented uterine cavity adhesions, demonstrating excellent biocompatibility and anti-adhesion efficacy [275]. In another study, a 3D-printed alginate scaffold loaded with the anti-VEGF drug bevacizumab (Avastin) significantly diminished the extent and strength of peritoneal adhesion bands in rats. The printed alginate/Avastin barrier reduced fibrosis and inflammation at the injury site by sustained local release of the drug, which lowered the pro-angiogenic and pro-inflammatory signals (VEGF, IL-6) involved in adhesion formation [273].

**Advantages:** Three-dimensional printing allows precise control over barrier geometry and the inclusion of cells or controlled-release drugs, enabling multifunctional “bioactive” adhesion barriers tailored to specific surgical sites.

**Limitations:** Bioprinting is technically complex and time-consuming, requiring specialized equipment and bioink formulations. Current 3D-printed anti-adhesion films are mostly in preclinical stages, and challenges remain in scaling up, sterilization, and regulatory approval for clinical use.

### 6.4. Layer-by-Layer Assembly (Multilayer Barrier Films)

Layer-by-layer (LbL) assembly involves depositing alternating layers of materials—often oppositely charged polymers or biomolecules—to build up a film with nanometer-scale control over thickness and composition. This technique can create multi-functional anti-adhesion barriers, for example by combining different polymer layers for desired surface properties and drug release profiles. One approach is polyelectrolyte multilayers; researchers demonstrated that coating alginate microgel cores with multiple LbL layers of polycations and polyanions can greatly prolong the release of small anti-inflammatory drugs, aiming to cover the critical 3–5 day period of post-surgery adhesion formation [276]. Increasing the number of bilayers on the drug-loaded particles extended drug release from about 1 day to up to 5 days, which could help modulate inflammation and fibrosis during early healing. Another strategy is constructing **bilayer or Janus films** where each side of the barrier has a different function. For example, a recent “dual-surface” membrane was made by merging a carboxymethyl cellulose (CMC) hydrogel layer with a collagen hydrogel layer through a vitrification process [276]. The resulting dry film (termed Bi-C-CVM) has a collagen-rich side that is slightly sticky, adhering to wet tissue, while the CMC side provides strong anti-adhesive properties. This design ensures the barrier stays in place (non-detachable) on the target organ while preventing adhesions on its outward-facing surface. In preclinical models, multi-layer barriers have shown improved performance. Researchers reported that an in situ, spray-fabricated, double-layer hydrogel (an inner healing-promotive alginate–gelatin layer topped with an anti-adhesive alginate–CMC layer) significantly reduced post-hepatectomy adhesion severity in rats, whereas either single layer alone was ineffective [277].

**Advantages:** LbL and multilayer fabrication offer exquisite control over film properties—one can integrate bioactive agents, create asymmetric surfaces, or program sequential degradation to match tissue healing. Such films can be highly conformable and tailored to various surgical scenarios.

**Limitations:** The assembly process can be labor-intensive and may involve many steps (dipping, drying, spraying), which is impractical for emergency use. Ensuring the uniformity and sufficient mechanical strength of ultra-thin multilayers can be difficult. Moreover, while several multilayer concepts show superior anti-adhesion effects in animal models [277], translating them into ready-to-use clinical products (with reasonable shelf-life and application methods) remains an ongoing challenge.

## 7. Conclusions and Future Perspectives

Postoperative adhesions remain a significant cause of morbidity following gynecologic and abdominal surgeries, despite advancements in surgical techniques. Biomaterial-based anti-adhesion barriers have shown great promise in mitigating adhesion formation by physically separating injured tissue surfaces during the critical phases of wound healing. Current clinically approved barriers are largely based on naturally derived polymers, such as hyaluronic acid, oxidized regenerated cellulose, and collagen. While these materials have demonstrated efficacy, their limitations—including poor mechanical strength, rapid degradation, and difficulty conforming to irregular anatomical sites—highlight the need for further innovation.

Recent research has broadened the biomaterials landscape with the incorporation of synthetic polymers, composite hydrogels, and bioengineered scaffolds. Novel fabrication strategies such as electrospinning, 3D bioprinting, melt electrowriting, and layer-by-layer assembly have enabled the creation of anti-adhesion films with tunable porosity, degradation kinetics, and mechanical properties. Moreover, the integration of bioactive agents—such as anti-inflammatory drugs, growth factors, and stem cells—into barrier systems is actively being explored to create multifunctional barriers that not only prevent adhesions, but also promote tissue regeneration and reduce inflammatory cascades. Studies using recombinant collagen, silk fibroin composites, decellularized extracellular matrices (dECM), and advanced alginate-based hydrogels have opened new avenues for developing biologically instructive anti-adhesion systems.

Looking forward, the design of next-generation anti-adhesion barriers will likely converge around several key principles. First, smart biomaterials capable of responding to the dynamic wound microenvironment—such as pH- or enzyme-responsive degradation—are highly desirable. These systems could allow the barrier to persist during the inflammatory phase but rapidly resorb once the risk of adhesion formation has passed, minimizing the foreign body load. Second, the integration of localized therapeutic delivery within the barrier structure offers substantial advantages. Anti-fibrotic agents, immunomodulators, or angiogenic factors could be embedded to further modulate healing processes favorably. Third, bioinspired designs mimicking native mesothelial surfaces (e.g., lubricity, anti-adhesive glycoprotein coatings) could enhance performance, ensuring that the biomaterial not only separates tissues mechanically, but actively resists cell attachment and fibrin deposition.

Personalized and patient-specific barriers may also become more prominent. With advances in 3D printing and imaging technologies, it is increasingly feasible to fabricate anti-adhesion membranes or patches customized to individual anatomical defects, ensuring better conformity and reducing complications associated with improper barrier placement. In parallel, cell-laden and tissue-engineered barriers—such as mesothelial cell-seeded scaffolds—represent an exciting frontier, particularly for complex injuries where simple mechanical separation is insufficient.

Despite these promising developments, several challenges remain. Regulatory pathways for multifunctional or living biomaterials are complex and will require robust preclinical validation. Long-term studies are needed to assess not only initial anti-adhesion efficacy, but also impacts on subsequent tissue remodeling, fertility (in gynecologic contexts), and chronic inflammation. Additionally, balancing manufacturability, shelf stability, and cost-effectiveness with advanced functionality will be crucial for clinical translation.

In summary, the future of postoperative anti-adhesion barriers lies at the intersection of materials science, bioengineering, and regenerative medicine. Emerging biomaterials that combine physical, biological, and therapeutic functions have the potential to dramatically improve patient outcomes after surgery. Continued interdisciplinary collaboration will be essential to realize the next generation of bioactive, responsive, and patient-tailored anti-adhesion barriers.

## Figures and Tables

**Table 1:** Summary of the advantages, disadvantages, and proposed solutions for using gelatin in anti-adhesion barriers. This table outlines gelatin’s key properties, such as biocompatibility, biodegradability, mechanical properties, and bioactivity, along with strategies to overcome limitations.

Aspect	Advantages	Disadvantages	Proposed Solutions	Ref.
**Biocompatibility**	Highly biocompatible, interacts well with tissues.	–	No issues, used widely for tissue scaffolding.	[53]
**Biodegradability**	Naturally degrades in the body, eliminating need for removal.	Degrades too quickly for long-term applications.	Crosslinking to control the degradation rate.	[54]
**Cell Affinity**	Supports cell attachment, proliferation, and differentiation.	–	No issues, ideal for promoting tissue regeneration.	[55]
**Mechanical Properties**	–	Poor mechanical strength, unsuitable for load-bearing tissues like bone and cartilage.	Crosslinking with agents or blending with stronger materials.	[51]
**Thermosensitivity**	Can form hydrogels at physiological temperatures, adaptable for various forms.	Sensitive to temperature, loses structure at body temperature.	Chemical modification or crosslinking for stability.	[56]
**Crosslinking Flexibility**	Can be chemically or physically crosslinked to enhance properties.	Toxicity risks from chemical crosslinkers (e.g., glutaraldehyde).	Use non-toxic methods like UV crosslinking or biocompatible agents.	[57]
**Cost and Availability**	Abundant, inexpensive, easy to source from animal collagen.	Batch-to-batch variability due to natural sources.	Standardize extraction and processing methods.	[58]
**Bioactivity**	Retains some bioactive motifs from collagen, supports cell signaling.	Limited bioactivity compared to native ECM.	Incorporation of additional bioactive molecules or growth factors.	[59]
**Processability**	Easy to process into films, sponges, or 3D-printed scaffolds.	Processing challenges with maintaining stability in vivo.	Optimize processing techniques, such as 3D printing or freeze drying.	[50]
**Immunogenicity**	Low immunogenicity when purified, reducing risk of immune reactions.	Potential immunogenicity from animal-derived sources.	Use synthetic, recombinant, or non-animal-derived gelatin.	[60]
**Hydrogel Formation**	Forms hydrogels, useful for drug delivery and wound healing.	–	No issues, effective in encapsulating bioactive agents.	[61]
**Drug/Growth Factor Delivery**	Excellent carrier for bioactive molecules, allowing controlled release.	–	No issues, widely used for therapeutic delivery systems.	[62]
**Nanotechnology Compatibility**	Compatible with nanoparticles, enhancing functionality or loaded drugs.	–	No issues, can be combined with advanced nanotechnology for applications.	[63]

**Table 2:** Common donor tissue sources for decellularized extracellular matrix (dECM), their species origin, and typical biomedical applications.

Tissue Source	Species Origin	Common Applications	Reference
**Small Intestinal Submucosa (SIS)**	Porcine	Hernia repair, anti-adhesion barriers, wound healing	[107]
**Urinary Bladder Matrix (UBM)**	Porcine	Soft tissue reconstruction, surgical mesh coatings	[108]
**Pericardium**	Bovine, porcine	Hernia repair, pelvic reconstruction	[109]
**Uterus**	Porcine, human (cadaveric)	Gynecologic tissue engineering, anti-adhesion scaffolds	[6,110]
**Amniotic Membrane**	Human (placental tissue)	Ocular repair, abdominal adhesion barriers	[111]

**Table 3:** Commercial CMC-based anti-adhesion barriers.

Product Name	Formulation	Application Route	Clinical Use Area	Key Strengths	Key Limitations
**Seprafilm^®^ (Genzyme Corporation, Deerfield, IL, USA)**	HA–CMC bioresorbable sheet	Open surgery (laparotomy)	General, colorectal, gynecologic	Well-studied; reduces adhesion severity; FDA-approved	Requires dry field; difficult laparoscopic application; possible foreign-body response
**SepraSpray^®^ (Genzyme Corporation, Cambridge, MA, USA** **)**	HA–CMC dual-powder spray	Laparoscopic and open	General abdominal, pelvic	Sprayable for MIS use; similar efficacy to Seprafilm	Variable coverage; mixed clinical results
**Interceed^®^ (Ethicon, Inc., Raritan, NJ, USA)**	Oxidized regenerated cellulose mesh	Open surgery	Gynecologic (e.g., myomectomy)	Easy to use; effective in hemostatic field	Inactivated by bleeding; not effective in contaminated areas
**Oxiplex^®^ (FzioMed, Inc., San Luis Obispo, CA, USA** **)/Intercoat^®^ (Intercoat Paints, Ltd., Walsall, UK** **)**	CMC–PEO hydrogel	Syringe (gel injection)	Gynecologic, abdominal, spinal	Conforms to irregular surfaces; favorable safety profile	Not FDA-approved in U.S.; moderate efficacy
**Guardix-SG^®^ (Hanmi Pharmaceutical Co., Ltd., Seul, Republic of Koreea** **)**	Thermosensitive poloxamer–alginate (CMC in early versions)	Laparoscopic and open	Gynecologic, thyroid, general	Thermogels at body temp; minimally invasive	Mostly used in East Asia; mixed data on long-term benefit

**Table 4:** Summary of differences between chitosan α, β, and γ.

Property	α-Chitosan	β-Chitosan	γ-Chitosan
**Chitin Source**	Exoskeleton of crustaceans	Squid pens, some marine species	Rare, mixed-source origins
**Polymer Alignment**	Anti-parallel	Parallel	Mixed (parallel and anti-parallel)
**Crystallinity**	High	Low	Moderate
**Solubility**	Low (acidic environment)	High (neutral pH)	Moderate
**Mechanical Strength**	High	Moderate	Intermediate
**Flexibility**	Low	High	Moderate
**Applications**	Scaffolds, wound healing, drug delivery	Hydrogels, wound care, drug delivery	Drug delivery, bioadhesives, anti-adhesion barriers

**Table 5:** Summary of PCL properties, processing methods, and modifications, including blending strategies to improve degradation and cytocompatibility for biomedical use.

Property	Description	Ref.
**Melting Temperature (Tm)**	~55–60 °C; allows easy processing and molding	[193,199,200,202,203,204,205,206]
**Glass Transition Temperature (Tg)**	~−60° C; remains flexible at room and rubbery at body temperature	
**Crystalline Behavior**	Semicrystalline; balance of crystalline and amorphous regions; affects melting and mechanical properties	
**Thermoplastic Behavior**	Can be melted and reshaped multiple times without significant degradation	
**Processing Techniques**	Extrusion, injection molding, electrospinning, 3D printing	
**Modification**	Blending with other polymers (e.g., PLA, PEG) to modify Tm, Tg, crystallinity, and degradation rate	
**Cytocompatibility**	Improved via blending with PLA, PEG, or surface modifications to enhance cell adhesion and reduce inflammatory response	
**Foreign Body Response**	Slow degradation may trigger encapsulation or chronic inflammation if not modified; blending can mitigate these effects	

**Table 6:** Summary of representative anti-adhesion barrier materials and their primary physiological elimination pathways. Routes include renal (urinary), hepatic (biliary), respiratory (as CO_2_), and lymphatic transport, depending on degradation profile and molecular size.

Material/Example	Primary Elimination Pathway	Notes
**Seprafilm^®^ (HA/CMC)**	Renal (urinary)	Cleared within 28 days via urine
**Low-MW HA (<12 kDa)**	Renal (urinary)	Only fragments < 12 kDa filtered
**PEG (<40 kDa)**	Renal (urinary)	Rapid clearance if <~40 kDa
**PEG (>50 kDa)**	Hepatic/biliary and renal	Captured by Kupffer cells; excreted in bile
**PLA/PLGA**	Respiratory (CO_2_) and renal	Lactic acid metabolized to CO_2_, glycolic acid to urine or CO_2_
**Oxidized Cellulose**	Respiratory (CO_2_) and renal	Degrades to glucose acid, enters metabolism
**Collagen/Gelatin**	Respiratory (CO_2_) or reused	Broken into amino acids; reused or oxidized
**Icodextrin (Adept^®^) (** **Baxter Healthcare Corporation, Singapore** **)**	Lymphatic → renal (after enzymatic breakdown)	Too large for blood absorption; enters via lymph, then urine

## Data Availability

No new data were created or analyzed in this study.
